# Deactivating mutations in the catalytic site of a companion serine carboxypeptidase-like acyltransferase enhance catechin galloylation in *Camellia* plants

**DOI:** 10.1093/hr/uhae343

**Published:** 2024-12-06

**Authors:** Xiangxiang Chen, Xue Zhang, Yue Zhao, Liping Gao, Zhihui Wang, Yanlei Su, Lingyun Zhang, Tao Xia, Yajun Liu

**Affiliations:** Department of Biological Sciences, School of Life Science, Anhui Agricultural University, No. 130, West Changjiang Road, Hefei 230036, Anhui, China; State Key Laboratory of Tea Plant Biology and Utilization, Anhui Agricultural University, No. 130, West Changjiang Road, Hefei 230036, Anhui, China; Tea Research Institute, Shandong Academy of Agricultural Sciences, No. 23788, Gongye North Road, Jinan 250100,Shandong, China; Department of Biological Sciences, School of Life Science, Anhui Agricultural University, No. 130, West Changjiang Road, Hefei 230036, Anhui, China; State Key Laboratory of Tea Plant Biology and Utilization, Anhui Agricultural University, No. 130, West Changjiang Road, Hefei 230036, Anhui, China; State Key Laboratory of Tea Plant Biology and Utilization, Anhui Agricultural University, No. 130, West Changjiang Road, Hefei 230036, Anhui, China; Department of Tea Science, College of Horticulture, South China Agricultural University, No. 483 Wushan Road, Guangzhou 510642, Guangdong, China; State Key Laboratory of Tea Plant Biology and Utilization, Anhui Agricultural University, No. 130, West Changjiang Road, Hefei 230036, Anhui, China; Department of Biological Sciences, School of Life Science, Anhui Agricultural University, No. 130, West Changjiang Road, Hefei 230036, Anhui, China; State Key Laboratory of Tea Plant Biology and Utilization, Anhui Agricultural University, No. 130, West Changjiang Road, Hefei 230036, Anhui, China

## Abstract

Galloylated flavan-3-ols are key quality and health-related compounds in tea plants of *Camellia* section *Thea*. *Camellia ptilophylla* and *Camellia sinensis* are two representative species known for their high levels of galloylated flavan-3-ols. Building on our knowledge of galloyl catechin biosynthesis in *C. sinensis*, we now focus on the biosynthesis of galloylated phenolics in *C. ptilophylla*, aiming to elucidate the mechanisms underlying the high accumulation of these compounds in *Camellia* species. The phenolic compounds in *C. ptilophylla* were identified and quantified using chromatographic and colorimetric methods. Genes involved in polyphenol galloylation were identified by correlating gene expression with the accumulation of galloylated phenolics across 18 additional *Camellia* species and one related species using Weighted Gene Coexpression Network Analysis. Key findings include the coexpression of SCPL4/2 and SCPL5 subgroup enzymes as crucial for galloylation of catechins, while SCPL3 and SCPL8 showed enzymatic activity related to hydrolyzable tannin synthesis. Variations in the amino acid sequences of SCPL5, particularly in the catalytic triad (T-D-Y vs S-D-H) observed in *C. ptilophylla* and *C. sinensis*, were found to significantly affect enzymatic activity and epigallocatechin gallate (EGCG) production. In conclusion, this research provides important insights into the metabolic pathways of *C. ptilophylla*, emphasizing the critical role of SCPL enzymes in shaping the phenolic profile within the section *Thea*. The findings have significant implications for the cultivation and breeding of tea plants with optimized phenolic characteristics.

## Introduction

Plant acyltransferases (ATs) play crucial roles in various plant metabolic pathways, particularly in the diverse phenolic metabolism pathways, contributing to the diversity of plant phenolic compounds [1]. They enhance the solubility, stability, transport, and storage of these molecules [2, 3], thus increasing plant tolerance to both biotic and abiotic stresses [4]. The enzymes involved in the acylation of plant phenolics mainly originate from two major acyltransferase families: BAHD acyltransferases (BAHD-ATs), which use acyl-CoA thioesters as acyl donors, and SCPL acyltransferases (serine carboxypeptidase-like proteins, SCPL-ATs), which utilize 1-*O*-*β*-D-glucose (*β*G) esters as acyl donors. SCPL-ATs belong to the serine carboxypeptidase (SCP) superfamily and represent a unique clade (Clade IA) with acyltransferase activity. To date, SCPL-ATs have been reported to participate in the synthesis of 14 acyl derivatives across ~10 species, including galloyl, sinapoyl, isobutyryl, feruloyl, and caffeoyl derivatives [5, 6]. Since the first SCPL-AT was discovered in tomato (*Lycopersicon pennellii*) [7], progress in identifying SCPL-AT enzyme functions has been slow. The precursor protein encoded by the *SCPL* gene needs to undergo a series of post-translational processing to form a catalytically active mature protein, which includes N-terminal signal peptide removal, glycosylation modifications, disulfide bond formation, proteolytic cleavage of internal spacer peptide, and formation of heterodimer [1, 8]. This complexity poses challenges for the functional characterization of SCPL-ATs and hampers a deeper understanding of their post-translational processing and catalytic mechanisms. To date, the functions of only ~10 plant SCPL-ATs have been characterized, including sinapoylcholine transferase (SCT), sinapoylanthocyanin transferase (SAT), sinapoylmalate transferase (SMT), and sinapoylsinapate transferase (SST) in *Arabidopsis thaliana* [5]. Additionally, two SCPL companion proteins work together to form the ECGT enzyme, which catalyzes the synthesis of galloyl catechins in *C. sinensis*.

Galloy catechin are galloylated products of flavan-3-ol and are a main class of flavan-3-ol derivatives in plants. These compounds are rich in numerous crops, such as tea plants, grape, and persimmon [9–11]. The well-known compound epigallocatechin gallate (EGCG) is rich in *C. sinensis*. This compound and its derivatives formed during processing impart a unique flavor to tea beverages. Interestingly, high levels of galloyl catechins are implicated in self-incompatibility mechanisms in *Camellia oleifera*, where they inhibit pollen tube growth during self-pollination but not cross-pollination, demonstrating their functional significance in plant reproduction [12]. More importantly, galloylated flavan-3-ols have multiple benefits to human health [13, 14]. Our previous research confirms that the coexpression of CsSCPL4 and CsSCPL5 in tea plants forms the ECGT enzyme, which catalyzes the biosynthesis of EGCG. CsSCPL4 was a catalytic acyltransferase with the conserved catalytic triad S-D-H and CsSCPL5 was a noncatalytic companion paralog of CsSCPL4 with a varied triad T-D-Y. CsSCPL5 assists in the post-translational processing of CsSCPL4. Notably, SCPL5 orthologs with a nonconservative catalytic triad are found in species like tea, persimmon, and grape, which are rich in high accumulations of galloylated flavanols. This raises the question of whether this variation is linked to the high levels of galloylated flavanols in the genus *Camellia* species. Building on previous research, this study focuses on *Camellia* species to investigate the role of SCPL5 in the biosynthesis of galloylated flavanols.

The genus *Camellia (C.)* is the largest in the Theaceae family, comprising a rich diversity of species with >200 identified, grouped into 20 sections [15, 16]. *Camellia* plants are highly valued for their applications in tea production, high-quality plant oil extraction, and ornamental uses in garden beds. They are also well-known for their accumulation of polyphenols. Our metabolomic analysis revealed that the accumulation of phenolic compounds in the fresh leaves of representative species in the genus *C.* can be categorized into three distinct types: galloyl catechin, hydrolyzable tannin, and proanthocyanidin accumulation. Notably, fresh leaves from *C.* sect. *Thea* exhibited significant galloyl catechin accumulation [17]. For example, cultivated tea plants (*C. sinensis*), which are representative of the galloyl catechin accumulation pattern, predominantly accumulate epimeric (*cis*) galloyl catechins such as EGCG and epicatechin gallate (ECG). In comparison, the fresh leaves of *C. ptilophylla* primarily accumulate *trans*-galloyl catechin gallocatechin gallate (GCG) [18, 19]. Drinking tea is known for its stimulating and refreshing effects, largely due to the high caffeine content in tea leaves. However, excessive tea consumption can lead to adverse effects such as insomnia and palpitations. *Camellia ptilophylla*, with its low caffeine content and high polyphenol levels, has garnered significant attention and is considered one of the most promising candidates for breeding and developing high-quality, caffeine-free tea plants. However, our understanding of phenolic accumulation and synthesis in this species is still limited. The specific accumulation of galloyl-type catechins in *Camellia* species is a focal point of our research, with particular emphasis on the synthesis of galloylated phenolic derivatives in *C. ptilophylla* in this work.

## Results

### Comprehensive profiling and comparative analysis of phenolic compound accumulation in *C. ptilophylla* and *C. sinensis*

*Camellia ptilophylla*, *C. sinensis*, and *C. oleifera* are three representative *Camellia* species, each accumulating different types of galloylated phenolic compounds. *Camellia ptilophylla*, *C. sinensis*, and *C. oleifera* accumulate galloylated *trans*-flavan-3-ols, galloylated *cis*-flavan-3-ols, and hydrolyzable tannins, respectively [20]. Previous studies identified the major phenolic compounds and their accumulation patterns in *C. sinensis* and *C. oleifera* leaves [21, 22]. In this study, we extend this analysis to *C. ptilophylla*, using mass spectrometry to identify and quantify its primary phenolic compounds.

**Figure 1:**
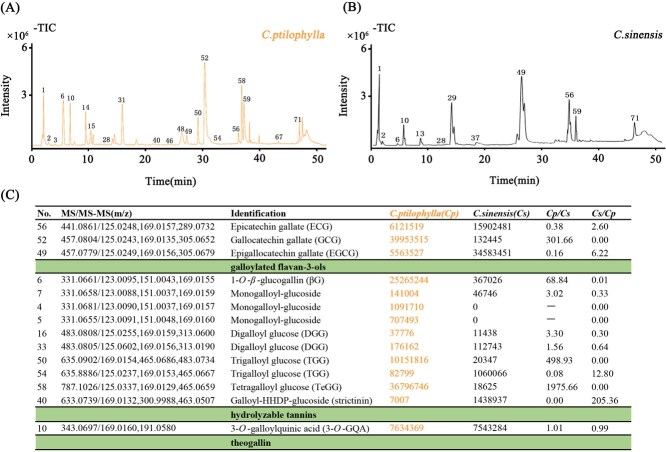
**Identification and comparative analysis of phenolic compounds in the leaves of *C. ptilophylla* and *C. sinensis* using UPLC-Q-TOF/MS/MS.** A and B. The TIC of the compounds in the leaf extract of *C. ptilophylla* and *C. sinensis* detected by UPLC-Q-TOF/MS/MS. The compound information is listed in Supplementary Table S1. C. Comparative analysis of the relative content of primary galloyl compounds based on mass spectrometry peak areas in leaf extracts of *C. ptilophylla* and *C. sinensis*.

Based on Ultra-high Performance Liquid Chromatography with Quadrupole Time-of-Flight Mass Spectrometry (UPLC-Q-TOF-MS/MS) technology, the total ion chromatogram (TIC) of the compounds in the methanol extract of *C. ptilophylla* was obtained as depicted in Fig. 1a. A total of 71 peaks were detected, and 66 compounds were identified, as detailed in Supplementary Table S1. Based on the retention times and mass spectrometry information of each compound, among the 66 identified compounds, there are 65 phenolic compounds and one amino acid compound (L-theanine). The phenolic compounds include 9 catechin compounds, 17 phenolic acid compounds, 6 hydrolyzable tannin compounds, 22 proanthocyanidin compounds, 10 flavanol compounds, and 1 other phenolic compound.

Quantification of phenolic compounds was carried out using Ultra-Performance Liquid Chromatography-Triple-Quadrupole Tandem Mass Spectrometry (UPLC-QqQ-MS/MS) with multiple reaction monitoring (MRM), based on the relative abundance of characteristic fragment ions. To better understand the relative content of major phenolics in fresh leaves of *C. ptilophylla*, we compared the peak areas of these compounds with those in fresh leaves of *C. sinensis*. The results are presented in Supplementary Table S2. Significant differences were observed in the types of accumulated phenolic compounds between *C. ptilophylla* and *C. sinensis* (Fig. 1a and b), particularly in the galloylated phenolics (Fig. 1c and Table S2). *Camellia ptilophylla* exhibited significantly higher levels of galloyl *trans*-catechins, specifically GCG, compared to *C. sinensis*, with GCG levels being 58-fold greater. In terms of hydrolyzable tannins, four monogalloyl-glucoside isomers were detected in *C. ptilophylla*, whereas only two were found in *C. sinensis*. The levels of all four monogalloyl-glucosides were notably higher in *C. ptilophylla*. Conversely, the complex hydrolyzable tannin strictinin accumulated in *C. sinensis* at levels 100-fold higher than in *C. ptilophylla*.

Additionally, *trans*-catechins and their derivatives, such as catechin (C), gallocatechin (GC), C-GC, and GC-GC, were significantly elevated in *C. ptilophylla*. The most abundant phenolic acid was 3-*O*-galloylquinic acid(3-*O*-GQA). The flavonol compound kaempferol-3-*O*-di-*p*-coumaroylhexoside (K-3-*O*-di-*p*-Cohex) was also presented at significantly higher levels in *C. ptilophylla* compared to *C. sinensis*.

Based on the standard curve for catechin compounds, the absolute content of catechins in both *C. ptilophylla* and *C. sinensis* was quantified (Fig. S1). The results showed that the primary catechins in *C. sinensis* are *cis*-catechins, with EGCG, ECG, and epigallocatechin at concentrations of 46.45, 22.84, and 15.31 mg/g, respectively. In contrast, the levels of these *cis*-catechins in *C. ptilophylla* were significantly lower, at 5.92, 6.32, and 0.98 mg/g. *Camellia ptilophylla* primarily accumulated *trans*-catechins, with GCG, C, and GC being the predominant forms at 38.94, 30.57, and 10.59 mg/g, respectively (Fig. S1). Conversely, in *C. sinensis*, the contents of GC and C were 1.67 and 2.88 mg/g, respectively, with GCG present in trace amounts. This comparison highlights the distinct catechin profiles of these two species, with *C. sinensis* primarily accumulating galloylated *cis*-catechins, while *C. ptilophylla* predominantly accumulates galloylated *trans*-catechins.

To further understand the characteristics of phenolic accumulation in *C. ptilophylla* and *C. sinensis*, the total content of several major types of phenolics was measured using colorimetric methods. The analysis revealed no significant difference in overall polyphenol content between the two species, with both averaging ~130 mg/g (Fig. S2a). However, the total flavan-3-ols content in *C. ptilophylla* was higher than that in *C. sinensis*, with values of 114 vs 94 mg/g, respectively (Fig. S2b). Additionally, the flavonol content in *C. ptilophylla* was significantly higher, at 2.08 mg/g, compared to 1.1 mg/g in *C. sinensis* (Fig. S2c) Additionally, the soluble proanthocyanidin (PA) content in *C. ptilophylla* was significantly higher, at 22 mg/g, compared to 4 mg/g in *C. sinensis* (Fig. S2d). These findings suggest that *C. ptilophylla* accumulates greater amounts of flavanols, flavonols, and soluble PAs than *C. sinensis*.

The accumulation patterns of these phenolic compounds in fresh leaves of *C. ptilophylla* at different developmental stages were further investigated. In young leaves and tender buds, catechins, most dimeric proanthocyanidins, gallic acid, and several of its derivatives—including monogalloyl-glucoside, DGG, and TGG—were found to accumulate at significantly higher levels. In contrast, their concentrations were relatively lower in mature leaves (Table S3).

### Screening of SCPLs related to biosynthesis of galloyl phenolic compounds in *C. ptilophylla*

**Figure 2:**
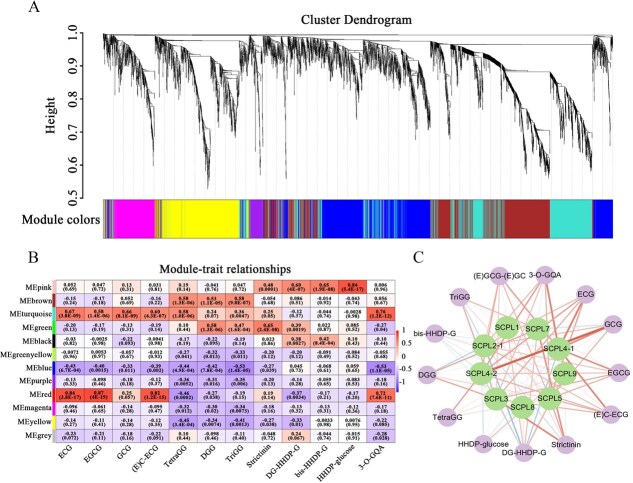
**Gene expression correlation analysis for identifying genes associated with galloylated phenolics biosynthesis in *Camellia* species.** A. Cluster dendrogram of DEGs based on WGCNA analysis. B. Module–trait relationships between galloylated phenolics accumulation and gene module expression. Major DEGs in each module are listed in Supplemental Data 2. The numbers in each cell represent the correlation and significance. C. Correlation between *SCPL* gene expression and galloylated phenolics accumulation based on Pearson analysis. The thickness of the lines represents the strength of the correlation. Correlation values are listed in Supplemental Data 3.

From the above analysis, it can be concluded that galloylated catechins are the predominant phenolic compounds in the fresh leaves of both *C. sinensis* and *C. ptilophylla*. To identify genes associated with the galloylation of phenolic compounds, we performed a Weighted Gene Coexpression Network Analysis (WGCNA) using the relative content of key phenolic compounds from fresh leaves of 19 *Camellia* species and their closely related species, along with all differentially expressed genes (DEGs). Data for this analysis were derived from previous studies [20]. The results indicated that the DEGs were classified into 12 gene expression modules (Fig. 2a and b). The accumulation of *cis*-galloylated catechins, such as ECG, EGCG, and EC-ECG, showed strong positive correlations with the red module, with correlation coefficients ranging from 0.82 to 0.87 (Fig. 2b). The turquoise module also demonstrated moderate correlations with *cis*-galloylated catechins, with coefficients between 0.58 and 0.67. The accumulation of *trans*-galloylated catechin GCG was more closely correlated with the turquoise module, with a coefficient of 0.66. Genes known to be involved in galloylated catechin biosynthesis, including *CHSa* (CSS0007714), *F3Ha* (CSS0016177), *F3’H* (CSS0048905), *CsUGT84A22* (CSS0032115), *SCPL4–1* (MSTRG.47639), *SCPL2–1* (CSS0019369), and *SCPL5* (CSS0041940), were positively correlated with both the red and turquoise modules (Supplemental Data 2). Additionally, the pink module was positively correlated with hydrolyzable tannin compounds, while galloylquinic acid accumulation was primarily associated with the turquoise module, followed by the red module (Fig. 2b).

Further Pearson correlation analysis was conducted to examine the expression of nine *SCPL* genes from the SCPL-IA family in *C. sinensis* and *C. ptilophylla* and their relationship with major galloylated phenolics. The results revealed a strong positive correlation between the expression of the *SCPL4–1* gene and the accumulation of ECG and EGCG, with correlation coefficients of 0.75 and 0.66, respectively. The expression of *SCPL4–2* showed an exceptionally high positive correlation with GCG accumulation, with a correlation coefficient of 0.96. Additionally, *SCPL3* showed correlations with hydrolyzable tannins, including strictinin and DGG, with coefficients of 0.70 and 0.51, respectively (Fig. 2c). These findings indicate that SCPL enzymes may be involved in the biosynthesis of various types of galloylated catechins.

### Functional characterization of the SCPL-IA gene family in *C. ptilophylla*

Based on the sequences of CsSCPL1 to CsSCPL9 identified in tea plants from previous studies [23], a local BLAST search was performed to identify homologous proteins annotated in the transcriptome of *C. ptilophylla* [24]. The SCPL proteins in *C. ptilophylla* were subsequently named based on sequence homology with the corresponding proteins in *C. sinensis* and their clustering in the phylogenetic tree (Fig. S3). In total, 10 SCPL-IA proteins were identified in *C. ptilophylla* and grouped into six distinct clades. The detailed protein sequence information is provided in Supplemental Data 5.

This study focuses on the biosynthesis of galloylated catechins in *C. ptilophylla*. The WGCNA analysis suggests that SCPL4 and SCPL5 may be involved in the synthesis of EGCG and GCG in *Camellia* plants. Within the SCPL2/4 subgroups of the phylogenetic tree, *C. ptilophylla* contains four sequences: CpSCPL4–1, CpSCPL4–2, CpSCPL2–1, and CpSCPL2–2, which correspond to orthologous sequences CsSCPL4 and CsSCPL2 in *C. sinensis*. Amino acid alignment reveals sequence identities of 97.29% between CpSCPL4–1 and CsSCPL4, 95.21%between CpSCPL4–2 and CsSCPL4, 99.18% between CpSCPL2–1 and CsSCPL2 (Supplemental Fig. S4). In the SCPL5 subgroup, *C. ptilophylla* contains two sequences: CpSCPL5–1 and CpSCPL5–2, with amino acid sequence identities of 98.33%. A key difference lies in the catalytic triad: while CpSCPL5–2 retains the conserved S-D-H triad, CpSCPL5–1 exhibits a variant T-D-Y triad.

Based on the nucleotide sequences of the *CpSCPL* genes screened from the transcriptome, primers were designed to clone the genes (Supplemental Data 1). Nine SCPL-IA genes were successfully cloned from *C. ptilophylla*, and their corresponding protein sequences are shown in Supplemental Data 5. The predicted molecular weights of these eight proteins ranged from 52 to 58 kDa, with isoelectric points between 4.9 and 5.9 (Supplemental Data 4). This data confirms the similarity in the protein characteristics of the SCPL-IA family.

**Figure 3:**
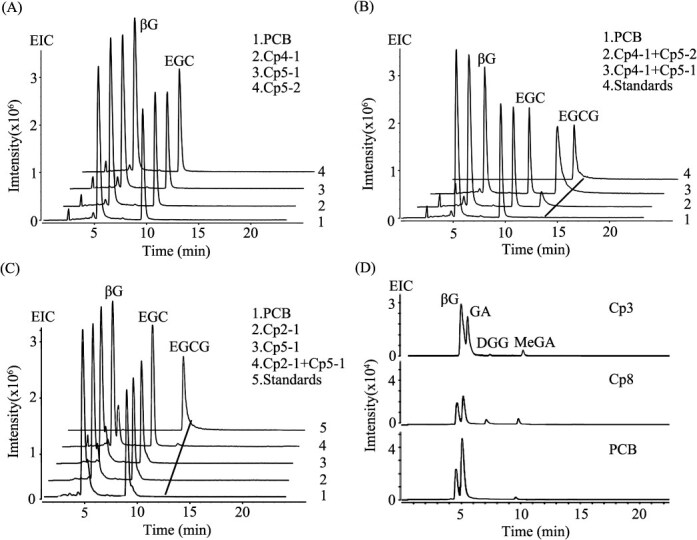
**Enzymatic activity analysis of recombinant SCPL enzymes from the SCPL-IA family in *C. ptilophylla.*** A and B. Enzyme activity analysis of proteins extracted from *N. benthamiana* leaves, expressed individually or in combination with *CpSCPL4–1*, *CpSCPL5–1*, and *CpSCPL5–2*. C. Enzyme activity analysis of recombinant proteins extracted from *N. benthamiana* leaves, coexpressing *CpSCPL2–1* with *CpSCPL5–1*. D. Enzyme activity analysis of proteins extracted from *N. benthamiana* leaves, individually expressing CpSCPL3 and CpSCPL8. MeGA, methyl gallate.

Previous research from our laboratory demonstrated that genes from the SCPL-IA family could not be expressed in a prokaryotic system, likely due to the system’s inability to perform the necessary post-translational modifications required by SCPL proteins [23]. Instead, a transient expression system in *Nicotiana benthamiana* proved effective for validating the enzymatic activity of SCPL-IA genes from *C. sinensis* and *C. oleifera* [21, 23]. Therefore, this system was employed to functionally validate the SCPL-IA genes from *C. ptilophylla*. Each of the 10 SCPL-IA family genes cloned from *C. ptilophylla* was transiently expressed in *N. benthamiana*. Crude enzyme extracts from the transiently overexpressed tobacco leaves were used to catalyze reactions with *β*G as the galloyl donor, and *β*G, EGC, and GC as acyl acceptors, to detect the enzymatic activity in producing galloylated catechins EGCG, GCG, and the hydrolyzable tannin DGG. The summarized enzyme activity results are presented in Supplementary Table S4. Notably, EGCG production was observed in the crude protein extracts from coexpression of CpSCPL2–1 + CpSCPL5–1, CpSCPL4–1 + CpSCPL5–1, and CpSCPL4–1 + CpSCPL5–2 (Fig. 3b and c). No enzymatic activity was detected when these SCPL proteins were expressed individually (Fig. 3a). However, individual expression of CpSCPL3 and CpSCPL8 demonstrated tannin hydrolase activity, leading to the biosynthesis of DGG (Fig. 3d and Table S4). Due to the absence of an effective method for purifying SCPL enzyme proteins for detailed kinetic analysis, a preliminary evaluation of SCPL enzyme activity was performed by comparing product yields under identical reaction conditions. The peak area of EGCG synthesized in crude enzyme extracts was 1.43 × 10^6^ for the coexpression of CpSCPL2–1 + CpSCPL5–1, 4.8 × 10^7^ for CpSCPL4–1 + CpSCPL5–1, and 8.3 × 10^6^ for CpSCPL4–1 + CpSCPL5–2. Among these, the coexpression of CpSCPL4–1 and CpSCPL5–1 produced the highest EGCG yield, followed by CpSCPL4–1 + CpSCPL5–2 (Table S5). Notably, no enzymatic activity was detected for the biosynthesis of GCG. The potential reasons for this lack of activity are analyzed in the discussion section.

### The impact of critical amino acids in SCPL5 on enzyme activity and *cis*-galloylated catechin synthesis in *C. ptilophylla*

**Figure 4:**
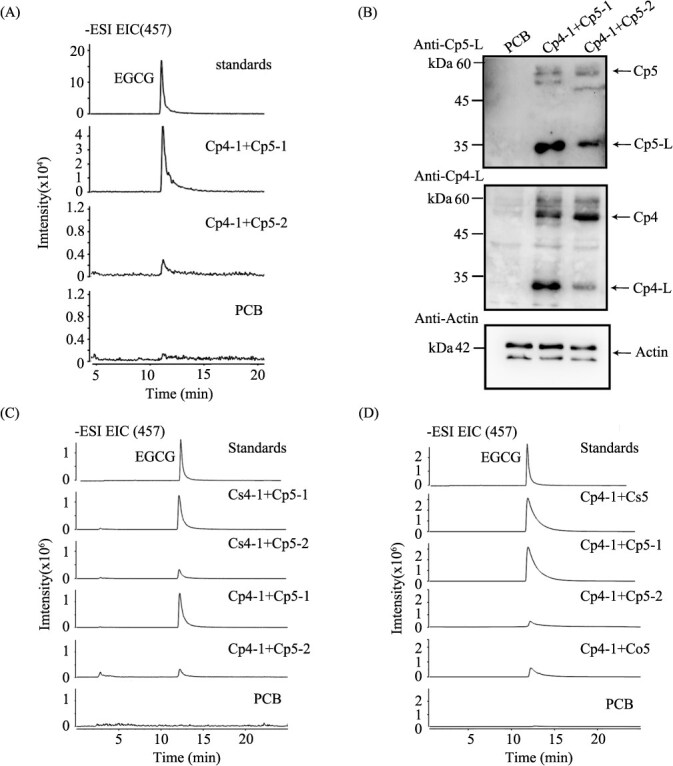
**The effect of the different SCPL5 on the enzymatic activity of the recombinant enzyme.** A. Recombinant proteins extracted from *N. benthamiana* leaves, coexpressing *CpSCPL4–1* with *CpSCPL5–1* or *CpSCPL4–1* with *CpSCPL5–2*, showed significant differences in enzymatic activity. B. Immunoblot analysis of recombinant proteins. C and D. Comparative analysis of enzyme activity of recombinant proteins extracted from *N. benthamiana* leaves coexpressing different SCPL4 and SCPL5 proteins from *C. sinensis* (Cs), *C. ptilophylla* (Cp), and *C. oleifera* (Co).

The enzyme activity assays revealed that the crude enzyme extracts from the coexpression of CpSCPL4–1 + CpSCPL5–1 produced significantly higher levels of EGCG compared to the coexpression of CpSCPL4–1 + CpSCPL5–2 (Fig. 3b). To investigate the cause of this disparity in enzyme activity, we conducted additional enzyme activity assays and western blot analyses on crude protein extracts from the transient coexpression of CpSCPL4–1 + CpSCPL5–1 and CpSCPL4–1 + CpSCPL5–2 in *N. benthamiana.* These analyses confirmed that while both combinations yielded enzyme products, the enzyme activity for CpSCPL4–1 + CpSCPL5–2 was markedly lower than that for CpSCPL4–1 + CpSCPL5–1 (Fig. 4a).

The SCPL4 and SCPL5 proteins from *C. sinensis* share very high sequence identity with those from *C. ptilophylla*, respectively (Supplemental Fig. S4). Consequently, antibodies against SCPL4 and SCPL5 from *C. sinensis* were used to immunoprecipitate the corresponding SCPL4 and SCPL5 proteins from *C. ptilophylla*. Western blot analysis revealed that the full CpSCPL5 protein (55 kDa) and the large subunit of CpSCPL5 (35 kDa) were both detected by the anti-CsSCPL5 antibody (Fig. 4b). Based on the post-translational processing characteristics of SCPL, these results indicate that both CpSCPL5–1 and CpSCPL5–2 were expressed in *N. benthamiana* and were processed into their corresponding mature proteins, including both large and small subunits. Similarly, two bands of the same molecular weight were detected by the anti-CpSCPL4 antibody, indicating that the CpSCPL4–1 gene was also expressed, translated, and processed correctly.

However, the band corresponding to the large subunit of SCPL4 and CpSCPL5 in the CpSCPL4–1 + CpSCPL5–2 sample was significantly weaker compared to that in the CpSCPL4–1 + CpSCPL5–1 sample (Fig. 4b). Our previous research has established that CsSCPL4 functions as a catalytic acyltransferase, while CsSCPL5 acts as a noncatalytic companion paralog (NCCP). The amino acid variation in CsSCPL5 not only impairs its own post-translational processing but also affects the processing of CsSCPL4 [23].

To further determine whether SCPL4 was responsible for the differences in enzyme activity, CsSCPL4–1 from *C. sinensis* and CpSCPL4–1 from *C. ptilophylla* were coexpressed with CpSCPL5–1 and CpSCPL5–2 from *C. ptilophylla*, respectively. The results indicated that the enzyme activity of CsSCPL4–1 coexpressed with CpSCPL5–1 was similar to that of CpSCPL4–1 with CpSCPL5–1, both significantly higher than that of CsSCPL4–1 with CpSCPL5–2 (Fig. 4c). To further assess the role of SCPL5 in enzyme activity differences, CpSCPL4–1 was coexpressed with CsSCPL5 (with the varied triad T-D-Y) from *C. sinensis*, CoSCPL5 (with the conserved catalytic triad S-D-H) from *C. oleifera*, and CpSCPL5–1 (with T-D-Y) and CpSCPL5–2 (with S-D-H). The results showed that the enzyme activity of CpSCPL4–1 coexpressed with CsSCPL5 and CpSCPL5–1 was significantly higher than that of CpSCPL4–1 coexpressed with CpSCPL5–2 and CoSCPL5 (Fig. 4d). Additionally, the activity of CpSCPL4–1 coexpressed with CpSCPL5–2 and CoSCPL5 in catalyzing the synthesis of EGCG was almost identical (Fig. 4d).

**Figure 5:**
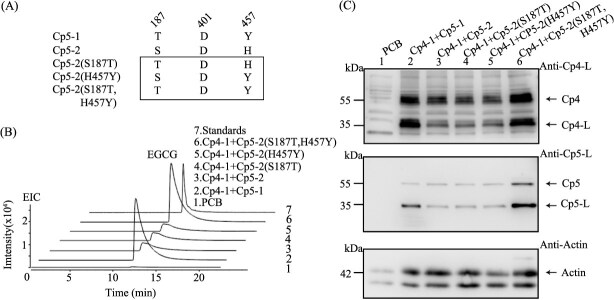
**The effect of site-directed mutation at catalytic triad of CpSCPL5 on enzymatic activity.** A. Schematic diagram of amino acid point mutations at catalytic triad of CpSCPL5. B. Effects of point mutations in the catalytic triad of CpSCPL5 on the activity of EGCG biosynthesis. C. The impact of point mutations on SCPL5 protein processing by immunoblot analysis.

To verify the critical residues of CpSCPL5 that influence enzyme activity based on the catalytic mechanism, three-point mutations were introduced into the catalytic triad residues of SCPL5–2. These mutants were designated as CpSCPL5–2(S187T), CpSCPL5–2(H457Y), and CpSCPL5–2(S187T, H457Y) (Fig. 5a). The enzyme assay showed that the enzyme activities of the crude protein extracts of the coexpression of CpSCPL4–1 + CpSCPL5–2(S187T), CpSCPL4–1 + CpSCPL5–2(H457Y), and CpSCPL4–1 + CpSCPL5–2 are significantly weaker than the enzyme activity of CpSCPL4–1 + CpSCPL5–1 and CpSCPL4–1 + CpSCPL5–2(S187T, H457Y). In comparison, the enzyme activity of the crude protein extracts of CpSCPL4–1 + CpSCPL5–2(S187T, H457Y) in synthesizing EGCG is relatively close to that of CpSCPL4–1 + CpSCPL5–1 (Fig. 5b). The point mutation enzyme activity results indicate that the mutation of T-D-Y of CpSCPL5 will affect the enzyme activity of the coexpression with CpSCPL4.

Immunoblotting analysis revealed the presence of the full-length CpSCPL4 and CpSCPL5 proteins, with a molecular weight of 55 kDa, along with their large subunit band at 35 kDa, in the samples of CpSCPL4–1 + CpSCPL5–1. Notably, the expression levels of both the 55 and 35 kDa proteins were significantly higher in the CpSCPL4–1 + CpSCPL5–1 and CpSCPL4–1 + CpSCPL5–2 (S187T, H457Y) samples compared to CpSCPL4–1 + CpSCPL5–2, CpSCPL4–1 + CpSCPL5–2 (S187T), and CpSCPL4–1 + CpSCPL5–2 (H457Y) (Fig. 5c). These immunoblot findings suggest that the T-D-Y catalytic triad of CpSCPL5 contributes to the stabilization of CpSCPL4, which in turn influences the overall enzyme activity in EGCG synthesis.

### Mutation of the catalytic site in SCPL5 correlates positively with the accumulation of galloylated catechins in *Camellia* species

**Figure 6:**
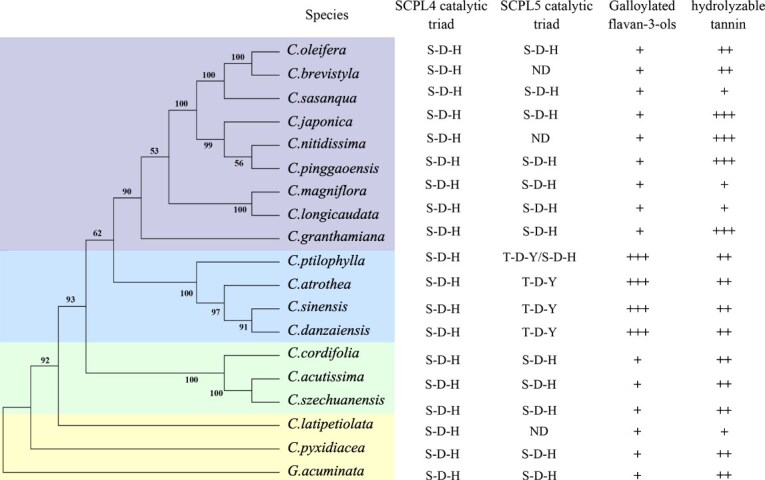
**Statistical analysis of amino acid mutations at catalytic sites of SCPL4 and SCPL5 and their association with the accumulation of gallic acid derivatives in *Camellia* species.** ‘+’ indicates the presence of the compound, with more ‘+’ signifying higher content.

To further elucidate the link between SCPL5 catalytic site mutations and the accumulation of galloylated catechins in *Camellia* species, we selected 18 species, along with 1 closely related species. These 18 species represent a broad cross-section of *C. Sect*. Phylogenetic analysis revealed that *Camellia* species could be divided into three subclades, with the four species from *Sect. Thea* clustering in a single branch (Fig. 6). We analyzed the protein sequences of SCPL4 and SCPL5 and relative quantification of gallic acid derivatives across these species. SCPL4 in all examined species consistently retains the S-D-H catalytic triad. However, in the transcriptomes of *Camellia nitidissima*, *Camellia brevistyla*, and *Camellia latipetiolata*, SCPL5 was not detected. In contrast, other species possessed the *SCPL5* gene, with species such *as Camellia szechuanensis*, *Camellia acutissima*, and *Camellia sasanqua* exhibiting SCPL5 with the conserved S-D-H triad. Notably, the four species from *Sect. Thea*, including *C. sinensis*, *Camellia danzaiensis*, and *Camellia atrothea*, harbored SCPL5 variants where the catalytic triad was replaced by T-D-Y, similar to the variation observed in *C. ptilophylla*, which expresses two SCPL5 isoforms: CpSCPL5–2 (S-D-H) and CpSCPL5–1 (T-D-Y) (Fig. 6).

Metabolomic analysis of phenolic compounds revealed the presence of galloylated catechins in all species, but higher concentrations were found specifically in *Sect. Thea* species, while other species contained minimal levels. The average content of galloylated catechins in *Sect. Thea* species was >100-fold higher than in other species (Supplemental Data 6). High levels of hydrolyzable tannins were detected in *Camellia granthamiana*, *Camellia japonica*, *Camellia pinggaoensis*, and *C. nitidissima*, with these species also showing distinct clustering based on their metabolite profiles in the phylogenetic tree (Fig. 6). Notably, *C. nitidissima*, which lacks SCPL5, also demonstrated a rich accumulation of hydrolyzable tannins, suggesting that the accumulation of these compounds in *Camellia* species may occur independently of SCPL5.

Furthermore, a noteworthy correlation was observed between high levels of galloylated catechins and the T-D-Y mutation in SCPL5 within *Sect. Thea*. This finding indicates that the coexpression of SCPL4 and SCPL5 proteins is closely associated with the galloylation process, with the T-D-Y mutation in SCPL5 correlating with the increased accumulation of galloylated catechins in *Camellia* species.

## Discussion

### Diversity and accumulation of phenolic compounds in *Camellia* species

The genus *Camellia*, the largest in the *Theaceae* family, comprises >200 species distributed across 18 sections and includes 220 described species, recognized for their ornamental value, tea production, and woody-oil uses, and has been cultivated globally for centuries [15, 16]. *Camellia* species are widely reported to contain phenolic compounds. *Camellia fascicularis*, an endemic plant in Yunnan province, China, is known for containing flavonols and phenolic acids [25]. Specifically, five flavonol compounds and four phenolic acids were identified in its leaves. Additionally, EGCG, gallic acid, and rutin have been isolated from *Camellia annamensis* [26]. In *C. japonica*, complex tannins, such as camelliatannins and camelliins, are abundantly present [27]. Furthermore, *Camellia pachyandra Hu.*, a species within the *Camellia* genus, has been found to contain 22 phenolic compounds, including 9 hydrolyzable tannins, 11 flavonol glycosides, and 2 simple phenolics [28]. Using LC–MS analysis, simple phenolic acids, flavonoids, and hydrolyzable tannins were detected across 18 representative *Camellia* species and one closely related species (Supplemental Data 6). Our previous results revealed distinct phenolic accumulation patterns in *Camellia* species, characterized by catechin-rich, proanthocyanidin-rich, and hydrolyzable tannin-rich accumulation types.

Section *Thea* in the genus *Camellia*, which is largely based on morphological comparison, includes 12 species [29]. Almost all species from the *Sect. Thea* have been used for making tea and consumed by the local people of its growing areas. Tea plants from *Sect. Thea* are particularly rich in phenolic compounds, with ~235 phenolic compounds reported to have clear structural formulas, of which 106 are galloyl derivatives [30]. Within this section, *C. sinensis* and *C. ptilophylla* primarily accumulate galloylated flavanols. Specifically, *C. sinensis* predominantly accumulates *cis*-galloylated catechins, such as EGCG and ECG, while *C. ptilophylla* primarily accumulates *trans*-galloylated catechins like GCG and CG [18, 19]. Mass spectrometry data shows that gallotannins such as TeGG are significantly more abundant in *C. ptilophylla* compared to *C. sinensis* (Fig. 1 and Table S2). Hydrolyzable tannins like monogalloyl glucose, DGG, TGG, and galloyl-hexahydroxydiphenoyl-glucose are detectable in high-quality fresh tea leaves harvested in early spring, making them indirect markers of tea quality [22, 31]. Galloylated catechins, which comprise 12%–17% of the dry weight of fresh tea leaves, are key determinants of tea quality, flavor, and health benefits [32].

### Acylation of *trans*- and *cis*-catechin in *Camellia* species

The acylation of plant phenolics is predominantly mediated by two major acyltransferase families: BAHD acyltransferases (BAHD-ATs), which use acyl-CoA thioesters as acyl donors, and SCPL acyltransferases (SCPL-ATs), which utilize *β*G esters as acyl donors [5]. However, exceptions exist, such as in *Echinacea purpurea* (Purple Coneflower), where an SCPL enzyme uses chlorogenic acid as an acyl donor and caftaric acid as an acyl acceptor to synthesize chicoric acid [6]. Current evidence suggests that SCPL enzymes play a key role in phenolic galloylation in *Camellia* plants (*C. sinensis*, *C. oleifera*, and *C. ptilophylla*), with *β*G esters acting as acyl donors [21, 23].

In *C. sinensis* and *C. ptilophylla*, SCPL enzymes from the SCPL-IA family, specifically SCPL4–1 and SCPL2, are known to catalyze the galloylation of *cis*-catechin [23]. In *C. sinensis*, SCPL4–2 can catalyze the acylation of *trans*-catechin GC to form GCG, although its activity is weak [20]. However, despite the high levels of GCG in *C. ptilophylla*, SCPL4–2 does not exhibit detectable GCG-synthesizing enzyme activity. Instead, it catalyzes the production of methyl gallate from methanol as a side reaction during the enzyme termination process (data not shown). Immunoassays confirm that SCPL4–2 undergoes proper post-translational processing, forming both large and small subunits, indicating that the protein is correctly assembled and functional. Interestingly, WGCNA correlation analysis has revealed a strong positive association between SCPL4–2 expression and GCG accumulation in *C. ptilophylla* (Fig. 2c), consistent with previous findings that SCPL4–2 (referred to as SCPL11–1 in the literature) is highly expressed in *C. ptilophylla* variants rich in GCG [33]. The absence of detectable GCG biosynthetic activity for SCPL4–2 in *C. ptilophylla* may have several underlying causes. First, alternative acyl donors may be involved in the reaction. Enzyme activity assays using galloylquinic acid, *β*G, TGG, and PGG as acyl donors did not reveal any activity (data not shown). It remains to be explored whether other conformations of monogalloyl glucose can serve as acyl donors. Notably, four monogalloyl glucose isomers are presented in *C. ptilophylla* (Supplementary Table S1 and S2). The biological roles of these isomers in the biosynthesis of galloyl phenolics within the plant is worth investigating. Second, SCPL4 may exist as multiple copies in the *C. ptilophylla* genome, producing highly similar transcripts. For instance, in *C. sinensis*, SCPL4 has at least five genomic copies that generate similar transcripts, but only CsSCPL4–1 and CsSCPL4–2 recombinants have shown detectable galloylated catechin-synthesizing activity [34]. However, in the Shuchazao genome, the transcripts CsSCPL4–1 and CsSCPL4–2 have not been annotated in the cDNA transcript sequences (TPIA2: http://tpia.teaplants.cn) [35]. The functions of other potential SCPL4 transcripts in *C. ptilophylla* remain to be validated. Lastly, the possibility that GCG synthesis involves other enzymes, such as BAHD family acyltransferases. For example, tea tannase exhibits both degalloylation and acylation activities [36]. It has been demonstrated that CsTA from *C. sinensis* can catalyze the formation of EGCG from *β*G and EGC. Additionally, TA is capable of catalyzing the synthesis of various galloyl phenolic compounds, including multiple isomers of DGG, a function not exhibited by SCPL3 [36]. We hypothesize that in *Camellia* species lacking SCPL5, the presence of EGCG may be attributed to the catalytic activity of TA. However, CsTA cannot catalyze the formation of GCG from *β*G and GC substrates. Further investigation is needed to determine whether TA enzyme can catalyze the biosynthesis of GCG using other acyl donors and GC acyl acceptor.

### Noncatalytic SCPL5 in galloylation pathways of phenolics in *Camellia* plants

The catalytic triad residues S-D-H of SCPL family enzymes is conserved and necessary for transferase activities [4]. In our previous work, we reported that two paralogous SCPL acyltransferases (SCPL-ATs) are responsible for the galloylation of flavan-3-ols: CsSCPL4, which retains the conserved S-D-H triad and functions as an active acyltransferase, and CsSCPL5, which contains a modified T-D-Y triad and acts as an NCCP [23]. The coexpression of CsSCPL4 and CsSCPL5 was responsible for the galloylation of *cis*-catechin. In *C. ptilophylla*, among the 10 SCPL-IA members, it was found that proteins from the SCPL4/2 subfamily require the presence of the SCPL5 subgroup paralogs, SCPL5–1 or SCPL5–2, as companions to assist in the post-translational modification and activation of SCPL4–1, SCPL4–2, and SCPL2 (Fig. 3). In contrast, SCPL3, derived from *C. oleifera* and *C. ptilophylla*, when expressed alone in *N. benthamiana*, was able to form an active recombinant enzyme that catalyzed the synthesis of simple hydrolyzable tannins (DGG, TGG, TeGG, and PGG) [21]. Similarly, SCPL8 from *C. ptilophylla*, when expressed alone, also formed an active recombinant enzyme capable of catalyzing the synthesis of DGG.

In our previous research, we investigated that tannin-rich plants typically contain pairs of SCPL4 and SCPL5 [23]. In this study, we observed that *C. nitidissima*, which lacks SCPL5, also demonstrates a rich accumulation of hydrolyzable tannins, suggesting that the accumulation of these compounds in *Camellia* species may occur independently of SCPL5 (Fig. 6). However, within *Camellia* species, the pairing of SCPL4 and SCPL5 is essential for the synthesis of EGCG. The data presented here indicates that SCPL5 with the variant triad (T-D-Y) is positively correlated with the accumulation of EGCG in *Camellia* species (Fig. 6). Immunoblot analysis showed that the mutated SCPL5–1 with the T-D-Y triad significantly enhanced the post-translational modification of the coexpressed SCPL4–1 protein, compared to the coexpression of SCPL4–1 and SCPL5–1 with the T-D-Y variant (Fig. 5). Despite these findings, the lack of crystal structure data for the interaction between the precursor forms of SCPL4–1 and SCPL5–1 makes it difficult to determine how mutations in the SCPL5 catalytic site affect post-translational modifications of both SCPL4–1 and SCPL5–1. This limitation hampers a full understanding of how mutations in SCPL5 modulate its own function and the function of its companion enzyme.

### Materials and methods

#### Plants and reagents

Fresh leaves of *Camellia* species were kindly supplied by the Jinhua International Camellia Species Garden (Zhejiang, China) and collected in April. Upon collection, the leaves were immediately frozen in liquid nitrogen, transported on dry ice, and stored at −80°C for subsequent analysis. For the gene transient expression experiments, leaves of *N. benthamiana* were cultivated in the rooftop climate chamber of the Biotechnology Building at Anhui Agricultural University. The chamber was maintained at a temperature of 24°C–26°C with a 16-h light and 8-h dark photoperiod.

The reagents used in this experiment included standard compounds such as catechin (C), epicatechin (EC), gallocatechin (GC), epigallocatechin (EGC), gallocatechin gallate (GCG), epigallocatechin gallate (EGCG), gallic acid (GA), and proanthocyanidin B2, all of which were purchased from Shanghai Yuanye Biotechnology Company. Rutin was obtained from Sigma-Aldrich, and 1-*O*-β-D-glucose (*β*G) was synthesized in-house by our laboratory.

#### Mass spectrometry analysis of phenolic compounds

The extraction of phenolic compounds in this study followed established protocols from previous research [11, 22]. Qualitative analysis was performed with an Agilent G6465A UPLC-Q-TOF-MS system in negative ion mode. Quantification was carried out using an Agilent 6460 QqQ-MS/MS LC system, also in negative ion mode, utilizing MRM for targeted analysis. The fragmentor voltage and collision energy parameters were optimized based on prior studies conducted in our lab [11, 22]. Chromatographic separation was performed on an Agilent 20RBAX RRHD Eclipse Plus C18 column. The mobile phase consisted of 0.4% acetic acid in water (Phase A) and acetonitrile (Phase B). The gradient elution commenced with 0.1% Phase B, increasing to 7% at 10 min, maintaining this level at 22 min. The Phase B concentration was then gradually raised to 11% at 25 min, 12% at 30 min, 14% at 31 min, and 35% at 43 min. At 45 min, Phase B was increased to 80%, before returning to 0.1% at 47 min, where it remained until the end of the run at 52 min. The flow rate was set at 0.2 ml/min, with a 5 μl injection volume, and the column temperature was maintained at 40°C. The mass spectrometry analysis was conducted using an electrospray ionization (ESI) source with a fragmentation voltage of 175 V, a sheath gas temperature of 350°C, and a flow rate of 11 l/min. The collision energy ranged from 15 to 40 eV, and the mass-to-charge ratio (m/z) was scanned from 79 to 1050.

#### SCPL gene screening of *C. ptilophylla* and bioinformatics analysis

Based on the transcriptome gene annotation of *C. ptilophylla* and comparative analysis of SCPL-IA family proteins in *C. sinensis* [23], the SCPL protein sequences from other species with an identity >75% were obtained through BLAST searches using the NCBI-Blast tool (https://www.ncbi.nlm.nih.gov/). Phylogenetic analysis of the identified SCPL-AT protein sequences was performed with MEGA version 6.0, applying the Neighbor-Joining method, and reliability was assessed through 1000 bootstrap replicates. The molecular weight, isoelectric point, and amino acid count for the SCPL-IA proteins in *C. ptilophylla* were predicted using an online protein molecular weight calculator (http://www.detaibio.com/sms2/protein_mw.html).

#### Site-directed mutagenesis in CpSCPL5

Gene-specific site mutation primers were designed targeting the catalytic triad and disulfide bond residues in the CpSCPL5 sequence. Using overlap extension polymerase chain reaction (PCR), we introduced site-specific nucleotide mutations to generate CpSCPL5–2(S187T), CpSCPL5–2(H457Y), and the double mutant CpSCPL5–2(S187T, H457Y). After verifying the mutations, the mutated cDNAs were inserted into the pCAMBIA1305 vector as previously described [23]. The primers used for site-directed mutations are listed in Supplemental Data 1.

#### Extraction of crude enzyme from *N. benthamiana* leaves

Five grams of frozen fresh *N. benthamiana* leaves were ground in 20 ml of chilled extraction buffer (100 mM phosphate buffer, pH 7.5, 150 mM NaCl) containing 0.5 g of water-insoluble polyvinylpolypyrrolidone (Solarbio Co., Ltd. Beijing, China) using a mortar. The homogenate was centrifuged at 10 000 *g* for 5 min, and the resulting supernatant was precipitated with ammonium sulfate (10%–80%). The precipitate was resuspended in 1 ml of extraction buffer. After a second centrifugation at 10 000 *g* for 5 min, the supernatant was placed into a dialysis bag and dialyzed against extraction buffer at 4°C for 8 h. The desalted protein extracts were quantified using a BCA assay kit (Jiancheng Bioengineering, Nanjing, China) and were then used for acyltransferase activity assays.

### Enzyme activity detection method

To assess the activity of the recombinant protein, crude protein from *N. benthamiana* leaves was utilized in enzyme activity assays, as follows: the reaction system had a total volume of 100 μl, which included 50 mM PBS (pH 6.0), 30 μg of protein, 0.4 mM acyl acceptor (EGC or GC), 0.4 mM acyl donor (*β*G), and 4 mM vitamin C. The reaction mixture was incubated at 30°C in a water bath for 3 h. Afterward, the reaction was stopped by briefly centrifuging the mixture and adding an equal volume of methanol, followed by vortexing and a 10-min room temperature incubation. The mixture was then sonicated for 5 min and centrifuged at 13 000 *g* for 10 min to remove precipitates. A 50-μl sample of the supernatant was transferred to a vial for analysis.

For the hydrolyzable tannin synthesis reaction, the reaction system was prepared with 50 mM PBS (pH 6.0), 30 μg of protein, 0.4 mM *β*G, and 4 mM vitamin C, and incubated at 40°C for 3 h. After brief centrifugation, the termination steps followed the same protocol as described above.

### Gene and metabolite coexpression network construction

To obtain a comprehensive understanding of the phenolic metabolism characteristics across *Camellia* species, we selected 1–4 representative species from each section (*Sect.*). The selected species include *C. atrothea*, *C. danzaiensis*, *C. ptilophylla*, and *C. sinensis* from *Sect. Thea (L.) Dyer*; *C. pinggaoensis* and *C. nitidissima* from *Sect. Chrysantha Chang*; *C. granthamiana* from *Sect. Protocamellia Chang*; *C. szechuanensis* from *Sect. Pseudocamellia Chang*; *C. acutissima* from *Sect. Theopsis Cob.St*; *Camellia pyxidiacea* from *Sect. Tuberculata Chang*; *Camellia cordifolia* from *Sect. Eriadria Coh.St*; *Camellia magniflora from Sect. Camellia.Dyer*, *C. japonica from Subsect. Lucidissima Chang*, *Camellia longicaudata* from *Sect. Longipedicellata Chang*; *C. oleifera* and *C. sasanqua* from *Sect. Oleifera Chang*; *C. brevistyla* from *Sect. Paracamellia Sealy*; and *C. latipetiolata* from *Sect. Furfuracea Chang*. One species related to the genus *Camellia*, *Gordonia acuminata*, was included. Tender leaves of these species in spring were collected for transcriptome sequencing and compound detection. WGCNA was conducted on 37 209 DEGs using the WGCNA shiny plugin in TBtools software. To construct a scale-free network, a soft-threshold power of 8 was selected after screening and scale-free topology structure detection. The minimum module size was set to 80 genes, and a merge cut height of 0.6 was used. The block size, corresponding to the number of genes remaining post-data cleaning, was set to 9072. The network and modules were constructed through the ‘blockwiseModules’ function, which allows for automated, unsupervised creation of the gene coexpression network. A signed, weighted correlation network was used to define the relationships between genes. The module eigengene, which reflects the first principal component of the scaled gene expression data within each module, was computed to summarize gene expression patterns. The module membership (kME) values, calculated through Pearson correlation, were used to assess the connectivity of genes within each module. The module networks were constructed with Cytoscape (v 3.7.1).

### Phylogenetic tree construction

A phylogenetic tree was constructed using high-quality 1:1 single-copy orthologous genes identified from the transcriptomic and genomic data of the 19 *Camellia* species mentioned above [17, 35]. To predict the coding sequence of each orthologous gene, we used TransDecoder (https://github.com/TransDecoder/TransDecoder) to identify open reading frames (ORFs) based on training sets derived from protein-coding genes, followed by annotation of Pfam domains using HMMER. The predicted ORFs were then individually aligned using MAFFT for further analysis [37]. After evaluating sequence consistency and conservation, orthologous gene alignments from each species were concatenated into a super gene sequence for phylogenetic analysis. The phylogenetic tree was built using the RAxML software package [38], and the optimal nucleotide substitution model was selected with ModelTest [39]. The resulting tree was visualized with MEGA, incorporating bootstrap support values from 1000 iterations [40].

### Statistical analysis methodology

Statistical analyses were performed using SPSS software (Version 16.0, SPSS Inc., Chicago). For comparisons between two groups, Student’s *t*-test was employed to evaluate statistical significance. The results are presented with significance levels indicated by one asterisk (*P < .05) for moderate significance, and two asterisks (**P < .01) for strong significance. All data represent the mean values from at least three independent biological replicates, ensuring the robustness of the results.

## Supplementary Material

Web_Material_uhae343

## Data Availability

All data generated from the study appear in the submitted article.
