# Supplementary material for: Deactivating mutations in the catalytic site of a companion serine carboxypeptidase-like acyltransferase enhance catechin galloylation in Camellia plants
Source: Hortic Res. 2024 Dec 6;12(3):uhae343. doi: 10.1093/hr/uhae343 (PMC11886809; doi:10.1093/hr/uhae343)
Supplement: Web_Material_uhae343 [file web_material_uhae343.zip › Supplementary methods.docx]

**Supplementary methods**

**Quantification of total polyphenols, flavonols, flavanols, and soluble proanthocyanidins**

Total polyphenol content in the samples was determined according to the national standard GB/T 8313-2018 with minor modifications. Specifically, 0.1 mL of the diluted phenolic extract was combined with 0.5 mL of diluted Folin-Ciocalteu reagent (0.1 mol/L). The mixture was vortexed and allowed to react at room temperature for 3–8 minutes. Subsequently, 0.4 mL of a 7.5% sodium carbonate (w/v) solution was added, and the mixture was incubated at room temperature for 1 hour. Absorbance was measured at 765 nm, using water as the blank. Total polyphenol content was quantified using a standard curve based on GA.

The total flavan-3-ol content in the samples was quantified using the vanillin-HCl method with slight modifications^1^. Specifically, 0.1 mL of diluted tea infusion or extract was mixed with 0.6 mL of 50% sulfuric acid containing 1% vanillin. The reaction mixture was allowed to stand at room temperature for 15 minutes. The absorbance was then measured at 500 nm, with a control group using deionized water instead of the tea extract. The flavonol content was calculated as follows: Flavonol absorbance=Treatment absorbance−Control absorbance. The total flavonol content was determined using a rutin standard curve.

Total flavonol content in the samples was determined according to the SN/T 4592-2016 standard, with minor modifications. Briefly, 0.5 mL of tea infusion or extract was added to a 2 mL centrifuge tube, followed by the addition of 0.5 mL of chloroform. The mixture was vortexed, then 0.5 mL of water was added and the mixture was gently centrifuged. The upper aqueous phase (0.5 mL) was transferred to a 4 mL centrifuge tube, where it was sequentially treated with 0.1 mL of aluminum nitrate (100 g/L) and 0.1 mL of potassium acetate (98 g/L). After vortexing, the volume was adjusted to 2 mL with water. The mixture was allowed to stand at room temperature for 1 hour. Absorbance was measured at 420 nm, with water as the blank. Flavonol content was calculated using the formula: Flavonol absorbance = Treatment absorbance − Control absorbance. Total flavonol content was quantified using a rutin standard curve.

The total soluble proanthocyanidin content in the samples was determined using the butanol-HCl cleavage method^2^. Specifically, 0.1 mL of tea infusion or extract was placed in a 2 mL cryogenic vial, and 0.6 mL of butanol-HCl reagent (95:5%, v/v) was added. The mixture was heated at 95°C in a water bath for 1 hour. The absorbance was measured at both 550 nm and 600 nm, using butanol-HCl reagent (95:5%, v/v) as the blank. The difference between the absorbances at 550 nm and 600 nm (OD550 - OD600) was calculated. The total soluble proanthocyanidin content was determined using a proanthocyanidin B2 standard curve. If the reaction mixture becomes turbid, chloroform may be added to clarify the solution by removing impurities, in accordance with the established method for anthocyanin extraction and detection in plant tissues^3^.

**Transcriptome sequencing method for plants**

cDNA libraries were sequenced on the Illumina platform by Genedenovo Biotechnology Co., Ltd (Guangzhou, China) with a sequencing depth of 6 G. The workflow was as follows: Total RNA was extracted using the Trizol reagent kit (Invitrogen, Carlsbad, CA, USA) according to the manufacturer's protocol. mRNA was then enriched from the total RNA using Oligo(dT) beads. To obtain high-quality clean reads, the reads were filtered using fastp (version 0.18.0), with rRNA-mapped reads removed. The remaining clean reads were used for assembly and gene abundance calculation. Two approaches were employed for gene assembly and annotation: 1) annotation against the *C. sinensis* reference genome of Shuchazao, and 2) de novo assembly using Trinity software to obtain transcript information from 19 plant species. For each transcriptional region, expression abundance and variations were quantified using FPKM (fragments per kilobase of transcript per million mapped reads), calculated with RSEM software^4^. Differential expression analysis of RNA was performed using DESeq2 software between two groups. Genes/transcripts with a false discovery rate (FDR) below 0.05 and an absolute fold change of ≥2 were considered differentially expressed^5^.

**Transient transformation expression in *N. benthamiana* and enzyme activity detection of SCPL**

The open reading frames (ORFs) of the *SCPL* genes were cloned into the CaMV35S promoter-driven PCB2004 binary vector using the Gateway LR Clonase enzyme (Invitrogen, Carlsbad, USA). The fusion plasmids were electroporated into *Agrobacterium tumefaciens* GV3101, spread onto Rif and Kan+ resistance plates, and incubated at 28 °C for 2-3 days. Single colonies were selected from the resistant medium and verified by colony PCR. After confirmation, positive colonies were cultured, and the suspensions were centrifuged. The pellets were resuspended in infiltration buffer (10 mM MES, pH 5.6, 10 mM MgCl₂, and 100 µM acetosyringone). The optical density at 600 nm (OD600) of the suspensions was adjusted to 0.6. For coexpression, equal volumes of the two suspensions were mixed and injected into the leaves of 30-day-old *N. benthamiana*. The infiltrated plants were grown in a greenhouse for 72 hours, after which the leaves were collected and frozen in liquid nitrogen for enzyme assays.

Reference：

1. Jiang Xiaolan,Hou Hua,Zhang Shuxiang *et al.*, Comparison of phenolic compound accumulation profiles in eight evergreen woody core eudicots indicating the diverse ecological adaptability of Camellia sinensis. *Scientia Horticulturae* 2017; **219**: 200-206.

2. Jiang X.,Liu Y.,Li W. *et al.*, Tissue-specific, development-dependent phenolic compounds accumulation profile and gene expression pattern in tea plant [*Camellia sinensis*]. *Plos One* 2013; **8**: e62315.

3. Liu Y. J.,Li M.,Li T. T. *et al.*, Airborne fungus-induced biosynthesis of anthocyanins in Arabidopsis thaliana via jasmonic acid and salicylic acid signaling. *Plant Science* 2020; **300**.

4. Li B.Dewey C. N., RSEM: accurate transcript quantification from RNA-Seq data with or without a reference genome. *BMC Bioinformatics* 2011; **12**: 323.

5. Love M. I.,Huber W.Anders S., Moderated estimation of fold change and dispersion for RNA-seq data with DESeq2. *Genome Biol* 2014; **15**: 550.
