# Supplementary material for: Deactivating mutations in the catalytic site of a companion serine carboxypeptidase-like acyltransferase enhance catechin galloylation in Camellia plants
Source: Hortic Res. 2024 Dec 6;12(3):uhae343. doi: 10.1093/hr/uhae343 (PMC11886809; doi:10.1093/hr/uhae343)
Supplement: Web_Material_uhae343 [file web_material_uhae343.zip › Supplimental_information.docx]

**Table S1. Qualitative analysis of phenolic compounds in *C.ptilophylla* leaves by Q-TOF-LC/MS**

| **Peak/no.** | **[M-H]− (m/z)** | **Time (min)** | **MS/MS(m/z)** | **Identification** |
| --- | --- | --- | --- | --- |
|  |  |  |  |  |
| 1 | 191.0575 | 1.345 | 127.0406,85.0302 | 1. (-)-quinic acid (QA) |
| 2 | 341.1108 | 1.449 | 179.0568 | Caffeic acid hexoside (CAH) |
| 3 | 173.0946 | 2.196 | 155.0834 | L-theanine |
| 4 | 331.0681 | 2.358 | 123.0090,151.0037,169.0157 | Monogalloyl-glucoside |
| 5 | 331.0655 | 3.716 | 123.0091,151.0048,169.0160 | Monogalloyl-glucoside |
| 6 | 331.0661 | 4.795 | 123.0095,151.0043,169.0155 | 1-*O*-*β*-glucogallin (βG) |
| 7 | 331.0658 | 5.584 | 123.0088,151.0037,169.0159 | Monogalloyl-glucoside |
| 8 | 169.0158 | 4.85 | 125.0249 | Galloyl acid (GA) |
| 9 | 191.0577 | 5.751 | 85.0301,127.0407 | D-(-)-quinic acid (QA) |
| 10 | 343.0697 | 5.981 | 169.0160,191.0580 | 3-*O*-galloylquinic acid (3-*O*-GQA) |
| 11 | 609.1222 | 6.94 | 125.0524,305.0676,423.0745 | (E)GC-(E)GC |
| 12 | 467.159 | 8.621 | 125.0205, 219.0667, 305.0662 | Gallocatechin-glucoside |
| 13 | 609.1332 | 8.842 | 125.0236, 305.0653,423.0720 | (E)GC-(E)GC |
| 14 | 305.0688 | 8.864 | 125.0253,179.0361,219.0682 | Gallocatechin (GC) |
| 15 | 593.1315 | 8.875 | 125.0260,289.0745,407.0819 | GC-C |
| 16 | 483.0808 | 11.24 | 125.0255,169.0159,313.0600 | Digalloyl glucose (DGG) |
| 17 | 897.1841 | 9.288 | 125.0238,425.0867,593.1266 | GC-C-GC |
| 18 | 593.1274 | 9.712 | 125.0239, 289.0736, 407.0773 | GC-C |
| 19 | 609.1266 | 10.732 | 125.0248,305.0700,423.0738 | (E)GC-(E)GC |
| 20 | 593.131 | 11.015 | 125.0255,289.0739,425.0896 | GC-C |
| 21 | 353.0892 | 11.24 | 135.0430, 179.0394,191.0901 | 3-caffeoylquinic acid |
| 22 | 881.1949 | 11.479 | 125.0243,289.0699,577.1319 | unknown |
| 23 | 897.1867 | 12.073 | 125.0240,303.0480,593.1287 | GC-C-GC |
| 24 | 881.1927 | 12.264 | 125.0239,289.0699,593.1264 | unknown |
| 25 | 577.1367 | 13.021 | 125.0240,289.579,425.0889 | Proanthocyanidin B1 |
| 26 | 337.095 | 13.283 | 119.0506,163.0409,191.0568 | 3-*p*-coumaroylquinic acid (3-*p*-CoQA) |
| 27 | 183.0325 | 13.373 | 78.0099,124.0177,168.0068 | Methyl gallic acid |
| 28 | 593.1316 | 13.679 | 125.0238,289.0709,407.0748 | GC-C |
| 29 | 305.0659 | 14.302 | 125.0257,167.0355,219.0662 | Epigallocatechin (EGC) |
| 30 | 761.1374 | 14.929 | 125.0244,305.0644,423.0695 | GCG-GC |
| 31 | 289.0745 | 15.413 | 125.0390,203.0697,245.0806 | Catechin (C) |
| 32 | 353.0695 | 16.066 | 191.0543 | Chlorogenic acid |
| 33 | 483.0805 | 16.868 | 125.0602,169.0156,313.0190 | Digalloyl glucose (DGG) |
| 34 | 353.0897 | 17.548 | 135.0456,173.0473,191.0570 | 4-caffeoylquinic acid (4-CQA) |
| 35 | 865.1977 | 18.436 | 125.0248,425.0867,577.1322 | (E)C-(E)C-(E)C |
| 36 | 1065.1934 | 18.565 | 313.0393,607.1030,895.1609 | unknown |
| 37 | 577.1376 | 19.265 | 125.0242,289.0701,425.0853 | Proanthocyanidin B3 |
| 38 | 745.1421 | 20.687 | 169.0124,305.0717,423.0706 | GCG-C |
| 39 | 1049.1978 | 21.899 | 125.0239,423.0685,591.1101 | unknown |
| 40 | 633.0739 | 21.883 | 169.0132, 300.9988, 463.0507 | Strictinin |
| 41 | 577.1330 | 22.398 | 125.0243,289.0706,425.0859 | Proanthocyanidin B4 |
| 42 | 337.0952 | 25.449 | 119.0502,173.0473,191.0555 | 5-*p*-coumaroylquinic acid (5-*p*-CoQA) |
| 43 | 577.1379 | 25.468 | 125.0247,289.0731,425.0888 | Proanthocyanidin B2 |
| 44 | 745.1413 | 25.516 | 125.0240,289.0702,407.0754 | GCG-C |
| 45 | 337.0946 | 25.553 | 119.0502,173.0472,191.0588 | 4-*p*-coumaroylquinic acid (4-*p*-CoQA) |
| 46 | 337.0946 | 25.81 | 119.0501,173.0329,191.0555 | cis-5-*p*-coumaroylquinic acid (cis-5-*p*-CoQA) |
| 47 | 289.0741 | 25.993 | 125.0231,203.0470,245.0485 | EC |
| 48 | 761.1381 | 26.142 | 125.0255,305.0696,423.0745 | GCG-GC |
| 49 | 457.0779 | 26.711 | 125.0249,169.0156,305.0679 | Epigallocatechin gallate (EGCG) |
| 50 | 635.0902 | 28.631 | 169.0154,465.0686,483.0734 | Trigalloyl glucose (TGG) |
| 51 | 729.1463 | 29.342 | 125.0259,289.0739,407.0771 | C-ECG |
| 52 | 457.0804 | 29.791 | 125.0243,169.0135,305.0652 | Gallocatechin gallate (GCG) |
| 53 | 745.1415 | 31.116 | 125.0247,305.0680,423.1290 | GCG-C |
| 54 | 635.8886 | 32.887 | 125.0237,169.015,3,465.0667 | Trigalloyl glucose (TGG) |
| 55 | 563.1419 | 33.93 | 353.0688,383.0785,473.1116 | Apigenin-6-C-glucosyl-8-C-arabinoside |
| 56 | 441.0861 | 35.027 | 125.0248,169.0157,289.0732 | Epicatechin gallate (ECG) |
| 57 | 441.0852 | 35.559 | 125.0260,169.0158,289.0731 | Catechin gallate (CG) |
| 58 | 787.1026 | 35.726 | 125.0337,169.0129,465.0659 | Tetragalloyl glucose (TeGG) |
| 59 | 609.0918 | 36.462 | 125.0242,287.0553,439.0648 | (E)GC-(E)GC |
| 60 | 463.0905 | 36.566 | 300.0293 | Quercetin-3-*O*-galactoside (Q-3-*O*-gal) |
| 61 | 433.0793 | 36.98 | 300.03 | unknown |
| 62 | 447.1114 | 37.214 | 227.0135,284.0810,285.0703 | Kaempferol-3-*O*-galactoside (K-3-*O*-gal) |
| 63 | 609.0893 | 37.496 | 125.0253,287.0557,439.0905 | (E)GC-(E)GC |
| 64 | 425.0890 | 37.758 | 125.0230, 169.0131, 273.0767 | Epiafzelechin gallate (EACG) |
| 65 | 781.2207 | 38.621 | 151.0035,285.0391,423.0728 | Kaempferol rhamnosyl rutinoside |
| 66 | 447.2241 | 41.532 | 284.0259, 285.0453 | Kaempferol-3-*O*-galactoside (K-3-*O*-gal) |
| 67 | 593.1316 | 41.955 | 125.0239,285.0399,327.0483 | Kaempferol-3-*O*-rutinoside (K-3-*O*-rut) |
| 68 | 447.2238 | 42.403 | 284.0350, 285.0422 | Kaempferol-3-*O*-glucosidea (K-3-*O*-glc) |
| 69 | 301.035 | 43.499 | 151.0024, 178.9976 | Quercetin (Q) |
| 70 | 285.0407 | 45.516 | 285.0407 | Kaempferol (K) |
| 71 | 739.1688 | 46.153 | 169.0135,285.0395,423.0696 | Kaempferol rhamnosyl rutinoside |
| **Note: The abbreviations of compound names are given in parentheses.** | | | | |

**Table S2. Comparative analysis of the relative content of phenolic compounds in *C.ptilophylla* and *C.sinensis* leaves (peak area)**

| Compound | Retention Time | | m/z ratio | | Peak area | | | |
| --- | --- | --- | --- | --- | --- | --- | --- | --- |
|  |  |  |  |  | *C.ptilophylla* | | *C.sinensis* | *Cp/ Cs* |
| C | | 15.16 | | 289-245 | | 399817 | 38082 | 10.50 |
| EC | | 25.063 | | 289-245 | | 35942 | 91205 | 0.39 |
| GC | | 9.053 | | 305-125 | | 408519 | 78881 | 5.18 |
| EGC | | 14.062 | | 305-125 | | 94412 | 397312 | 0.24 |
| ECG | | 35.477 | | 441-169 | | 477501 | 1766349 | 0.27 |
| EGCG | | 26.435 | | 457-169 | | 1328874 | 10235283 | 0.13 |
| GCG | | 29.634 | | 457-169 | | 8393126 | 142809 | 58.77 |
| Sum | |  | |  | | 11138191 | 12749921 | 0.87 |
| Prothocyanidin B1,B3 | | 14.032 | | 577-289 | | 22827 | 10954 | 2.08 |
| Prothocyanidin B4 | | 18.287 | | 577-289 | | 69968 | 112394 | 0.62 |
| Prothocyanidin B2 | | 20.929 | | 577-289 | | 11064 | 31023 | 0.36 |
| (E)GC-(E)C | | 10.087 | | 593-289 | | 273965 | 849 | 322.69 |
| (E)GC-(E)C | | 13.792 | | 593-289 | | 49976 | 7364 | 6.79 |
| C-GC | | 11.034 | | 593-305 | | 35250 | 975 | 36.15 |
| C-EGC | | 12.921 | | 593-305 | | 4203 | 12832 | 0.33 |
| GC-GC | | 8.385 | | 609-423 | | 121197 | 2128 | 56.95 |
| (E)GC-(E)GC | | 10.383 | | 609-423 | | 15294 | 23249 | 0.66 |
| C-ECG | | 29.175 | | 729-407 | | 6927 | 19402 | 0.36 |
| EC-ECG | | 30.63 | | 729-407 | | 5527 | 23491 | 0.24 |
| (E)GCG-(E)GC | | 14.746 | | 761-423 | | 21730 | 21609 | 1.01 |
| Sum | |  | |  | | 637928 | 266270 | 2.40 |
| Galloyl acid | | 5.286 | | 169-125 | | 41584 | 28673 | 1.45 |
| Methyl gallic acid | | 13.708 | | 183-124 | | 147104 | 462271 | 0.32 |
| βG | | 4.667 | | 331-169 | | 518652 | 158483 | 3.27 |
| Monogalloyl-glucoside | | 5.344 | | 331-169 | | 37898 | 17782 | 2.13 |
| Monogalloyl-glucoside | | 2.747 | | 331-169 | | 29219 | 0 | — |
| Monogalloyl-glucoside | | 3.865 | | 331-169 | | 42599 | 0 | — |
| TGG | | 28.405 | | 635-169 | | 306430 | 1626 | 188.46 |
| TGG | | 32.337 | | 635-169 | | 8513 | 183995 | 0.05 |
| TeGG | | 36.174 | | 787-169 | | 650736 | 695 | 936.31 |
| DGG | | 16.13 | | 483-271 | | 60739 | 92913 | 0.65 |
| DGG | | 12.599 | | 483-271 | | 4521 | 16141 | 0.28 |
| HHDP-galloylglucoside(Strictinin) | | 17.807 | | 633-301 | | 24838 | 2484626 | 0.01 |
| Sum | |  | |  | | 1872833 | 3447205 | 0.54 |
| D-(-)-quinic acid | | 2.099 | | 191-85 | | 99108 | 90489 | 1.10 |
| 3-*O*-GQA | | 6.061 | | 343-191 | | 1328611 | 1810327 | 0.73 |
| 5-*p*-CoQA | | 19.873 | | 337-173 | | 65718 | 104086 | 0.63 |
| cis-5-*p*-CoQA | | 24.556 | | 337-173 | | 365723 | 384596 | 0.95 |
| 3-*O*-CQA | | 11.082 | | 353-191 | | 18756 | 5697 | 3.29 |
| 5-*O*-CQA | | 15.332 | | 353-191 | | 86013 | 44832 | 1.92 |
| 4-*O*-CQA | | 17.144 | | 353-191 | | 43782 | 3183 | 13.75 |
| 3-*p*-CoQA | | 13.742 | | 337-163 | | 373312 | 44163 | 8.45 |
| CAH | | 2.184 | | 341-89 | | 32757 | 45444 | 0.72 |
| Sum | |  | |  | | 2413780 | 2532817 | 0.95 |
| K | | 45.319 | | 285-285 | | 13429 | 4371 | 3.07 |
| Q | | 39.826 | | 301-151 | | 431 | 425 | 1.01 |
| K-3-*O*-gal | | 37.463 | | 447-284 | | 4938 | 96554 | 0.05 |
| K-3-*O*-glc | | 38.085 | | 447-284 | | 28333 | 27730 | 1.02 |
| Q-3-*O*-gal | | 35.956 | | 463-300 | | 39579 | 74081 | 0.53 |
| M-3-*O*-gal | | 32.406 | | 479-316 | | 9559 | 204779 | 0.05 |
| M-3-*O*-glu | | 33.183 | | 479-316 | | 9098 | 89579 | 0.10 |
| K-3-*O*-rut | | 37.584 | | 593-285 | | 64311 | 52003 | 1.24 |
| Q-3-*O*-rut | | 35.601 | | 609-300 | | 21971 | 4179 | 5.26 |
| Q-3-*O*-rut | | 35.938 | | 609-300 | | 18975 | 6434 | 2.95 |
| M-3-*O*-rut | | 32.94 | | 625-316 | | 1786 | 22966 | 0.08 |
| K-3-*O*-galactosylrutinoside | | 35.993 | | 755-285 | | 1557 | 208730 | 0.01 |
| Q-3-*O*-glucosylrutinoside | | 35.185 | | 771-301 | | 401 | 17184 | 0.02 |
| M-3-*O*-galactosylrutinoside | | 30.887 | | 787-316 | | 46 | 17445 | 0.00 |
| K-3-*O*-di-*p*-coumaroylhexoside | | 46.121 | | 739-285 | | 374780 | 101561 | 3.69 |
| Sum | |  | |  | | 589194 | 928021 | 0.63 |
| Note：Quantification of phenolics was performed using UPLC-QqQ-MS/MS with multiple reaction monitoring (MRM), based on characteristic fragment ion abundance in both positive and negative ion modes. Compound abbreviations are consistent with those used in Table S1. | | | | | | | | |

**Table S3. Relative quantitative analysis of phenolic compounds in leaves of *C.ptilophylla* at different developmental stages (peak area)**

| Classification | Compound | m/z ratio | | Peak area | | | | | | |
| --- | --- | --- | --- | --- | --- | --- | --- | --- | --- | --- |
|  |  |  |  | Bud | | | First leaf | | Second leaf | Third leaf |
|  | C | 289-245 | | 593230 | | | 529744 | | 322705 | 341652 |
|  | EC | 289-245 | | 51230 | | | 11645 | | 4060 | 3891 |
|  | GC | 305-125 | | 472824 | | | 424131 | | 12806 | 10493 |
|  | EGC | 305-125 | | 11866 | | | 2391 | | 1015 | 1320 |
| Catechins | EACG | 425-273 | | 42832 | | | 9368 | | 254 | 250 |
|  | ECG | 441-169 | | 616238 | | | 250599 | | 6522 | 5186 |
|  | EGCG | 457-169 | | 777048 | | | 174084 | | 7882 | 4714 |
|  | GCG | 457-169 | | 7843246 | | | 8622874 | | 86691 | 51041 |
|  | GC-glc | 467-305 | | 2704 | | | 4775 | | 0 | 0 |
|  | Galloyl acid | 169-125 | | 19078 | | | 27882 | | 270 | 350 |
|  | D-(-)-quinic acid | 191-85 | | 65370 | | | 59142 | | 274565 | 298379 |
|  | D-(-)-quinic acid | 191-85 | | 70067 | | | 75220 | | 0 | 0 |
|  | Monogalloyl-glucoside | 331-169 | | 48307 | | | 45272 | | 22 | 28 |
|  | Monogalloyl-glucoside | 331-169 | | 36134 | | | 33614 | | 0 | 594 |
| Phenolic acids and their derivatives | βG | 331-169 | | 574010 | | | 582223 | | 826 | 58 |
|  | Monogalloyl-glucoside | 331-169 | | 1395 | | | 1903 | | 0 | 0 |
|  | 3-*p*-coumaroylquinic acid | 337-163 | | 7491 | | | 32070 | | 1698845 | 1554524 |
|  | 5-*p*-coumaroylquinic acid | 337-173 | | 2815 | | | 58383 | | 13026 | 15672 |
|  | cis-5-*p*-coumaroylquinic acid | 337-173 | | 293446 | | | 328208 | | 24452 | 28275 |
|  | Caffeic acid hexoside | 341-89 | | 35681 | | | 32448 | | 9157 | 8291 |
|  | 3-O-galloylquinic acid | 343-191 | | 1314504 | | | 1158294 | | 171 | 35 |
|  | 3-*O*-caffeoylquinic acid | 353-191 | | 3746 | | | 13082 | | 6468 | 7114 |
|  | 5-*O*-caffeoylquinic acid | 353-191 | | 4086 | | | 50155 | | 2745 | 1312 |
|  | 4-*O*-caffeoylquinic acid | 353-191 | | 215346 | | | 34105 | | 142 | 677 |
|  | DGG | 483-271 | | 3188 | | | 3453 | | 0 | 0 |
|  | DGG | 483-271 | | 336 | | | 405 | | 0 | 0 |
|  | DGG | 483-271 | | 2671 | | | 3287 | | 0 | 0 |
|  | DGG | 483-271 | | 17785 | | | 29005 | | 0 | 0 |
| Hydrolysable tannins | DGG | 483-271 | | 4805 | | | 7282 | | 0 | 0 |
|  | Strictinin | 633-301 | | 411 | | | 367 | | 302 | 399 |
|  | TGG | 635-169 | | 167383 | | | 214314 | | 441 | 146 |
|  | TGG | 635-169 | | 18989 | | | 30018 | | 481 | 88 |
|  | TGG | 635-169 | | 4455 | | | 9958 | | 250 | 60 |
|  | TeGG | 787-169 | | 494694 | | | 558605 | | 7308 | 3955 |
|  | Prothocyanidin B1 | 577-289 | | 17073 | | | 9080 | | 45067 | 39163 |
|  | Prothocyanidin B3 | 577-289 | | 160624 | | | 143957 | | 152319 | 153064 |
|  | Prothocyanidin B4 | 577-289 | | 63997 | | | 35590 | | 5334 | 1703 |
| Proanthocyanidins | Prothocyanidin B2 | 577-289 | | 6196 | | | 4270 | | 1577 | 2153 |
|  | (E)GC-(E)C | 593-289 | | 235159 | | | 240059 | | 3484 | 3520 |
|  | (E)C-(E)GC | 593-305 | | 35132 | | | 27272 | | 2125 | 2259 |
|  | (E)GC-(E)GC | 609-423 | | 2728 | | | 1584 | | 512 | 498 |
|  | (E)GC-(E)GC | 609-423 | | 77791 | | | 78935 | | 577 | 387 |
|  | (E)GC-(E)GC | 609-423 | | 11863 | | | 11102 | | 0 | 0 |
|  | (E)C-ECG | 729-407 | | 24705 | | | 4843 | | 0 | 0 |
|  | (E)C-ECG | 729-407 | | 2412 | | | 975 | | 0 | 0 |
|  | (E)GC-ECG | 745-407 | | 29623 | | | 9967 | | 0 | 0 |
|  | (E)GCG-(E)GC | 761-423 | | 16904 | | | 8281 | | 108 | 67 |
| Flavones and Their Glycosides | A-6-C-glc-8-C-ara | | 563-353 | | 9 | 10 | | 1580 | | 1743 |
|  | A-6-C-ara-8-C-ara | | 563-353 | | 31 | 40 | | 4656 | | 4555 |
|  | Rhamnosylvitexin | | 577-293 | | 23 | 29 | | 742 | | 571 |
|  | Vitexin-2"-O-rha | | 577-293 | | 12 | 14 | | 690 | | 933 |
|  | K | | 285-285 | | 27161 | 21792 | | 16316 | | 14242 |
|  | K-3-*O*-glc | | 447-285 | | 3482 | 8266 | | 19428 | | 16379 |
|  | Quercetin-3-*O*-glu/gal | | 463-300 | | 456 | 935 | | 130376 | | 133424 |
|  | Quercetin-3-*O*-glu/gal | | 463-300 | | 1146 | 2323 | | 104466 | | 107298 |
| Flavonols and Their Glycosides | Myricetin-3-*O*-glu/gal | | 479-316 | | 2602 | 3983 | | 2416 | | 1646 |
|  | Myricetin-3-*O*-glu/gal | | 479-316 | | 3131 | 8384 | | 3050 | | 2274 |
|  | kaempferol-3-*O*-rutinoside | | 593-285 | | 34615 | 59232 | | 9857 | | 7123 |
|  | Quercetin-3-*O*-rutinoside | | 609-300 | | 6484 | 8777 | | 192970 | | 211231 |
|  | Myricetin-3-*O*-rutinoside | | 625-316 | | 863 | 1150 | | 758 | | 644 |
|  | K-3-*O*-di-*p*-Cohex | | 739-285 | | 455299 | 281067 | | 4980 | | 3075 |
|  | kaempferol rhamnodiglucoside | | 755-285 | | 162 | 116 | | 216 | | 239 |
|  | kaempferol rhamnodiglucoside | | 755-285 | | 439 | 875 | | 345 | | 344 |
| Note：Quantification of phenolics was performed using UPLC-QqQ-MS/MS with multiple reaction monitoring (MRM), based on characteristic fragment ion abundance in both positive and negative ion modes. Compound abbreviations are consistent with those used in Table S1. | | | | | | | | | | |

**Table S4. Enzyme activity validation of CpSCPL recombinant proteins expressed in leaves of *N. benthamiana***

|  | **EGCG** | **GCG** | **DGG** |
| --- | --- | --- | --- |
| SCPL1 | － | － | － |
| SCPL2-1 | － | － | － |
| SCPL2-2 | － | － | － |
| SCPL3 | － | － | ++ |
| SCPL4-1 | － | － | － |
| SCPL4-2 | － | － | － |
| SCPL5-1 | － | － | － |
| SCPL5-2 | － | － | － |
| SCPL6 | － | － | － |
| SCPL8 | － | － | ＋ |
| SCPL1+SCPL5-1 | － | － | － |
| SCPL1+SCPL5-2 | － | － | － |
| SCPL2-1+SCPL5-1 | ＋ | － | － |
| SCPL2-1+SCPL5-2 | + | － | － |
| SCPL2-2+SCPL5-1 | － | － | － |
| SCPL2-2+SCPL5-2 | － | － | － |
| SCPL4-1+SCPL5-1 | ++ | － | － |
| SCPL4-1+SCPL5-2 | ＋ | － | － |
| SCPL4-2+SCPL5-1 | － | － | － |
| SCPL4-2+SCPL5-2 | － | － | － |
| SCPL6+SCPL5-1 | － | － | － |
| SCPL6+SCPL5-2 | － | － | － |
| SCPL8+SCPL5-1 | － | － | + |
| SCPL8+SCPL5-2 | － | － | + |

**Note:** "+" indicates enzyme activity detected, with more "+" signs indicating higher enzyme activity. "-" indicates no activity detected.

| **Table S5. The quantity (measured as peak area) of both the substrate and the product was assessed in the EGCG biosynthesis reaction** | | | | | | | | |
| --- | --- | --- | --- | --- | --- | --- | --- | --- |
| peak area | PCB2004 | Cp2-1 | Cp4-1 | Cp5-1 | Cp5-2 | Cp2-1+ Cp5-1 | Cp4-1+ Cp5-1 | Cp4-1+ Cp5-2 |
| βG | 7.57×10^7^ | 7.52×10^7^ | 7.20×10^7^ | 7.10×10^7^ | 7.20×10^7^ | 7.57×10^7^ | 7.57×10^7^ | 7.57×10^7^ |
| EGC | 4.70×10^7^ | 4.75×10^7^ | 3.70×10^7^ | 3.80×10^7^ | 3.80×10^7^ | 7.57×10^7^ | 7.57×10^7^ | 7.57×10^7^ |
| EGCG | 0 | 0 | 0 | 0 | 0 | 1.43×10^6^ | 4.80×10^7^ | 8.30×10^6^ |


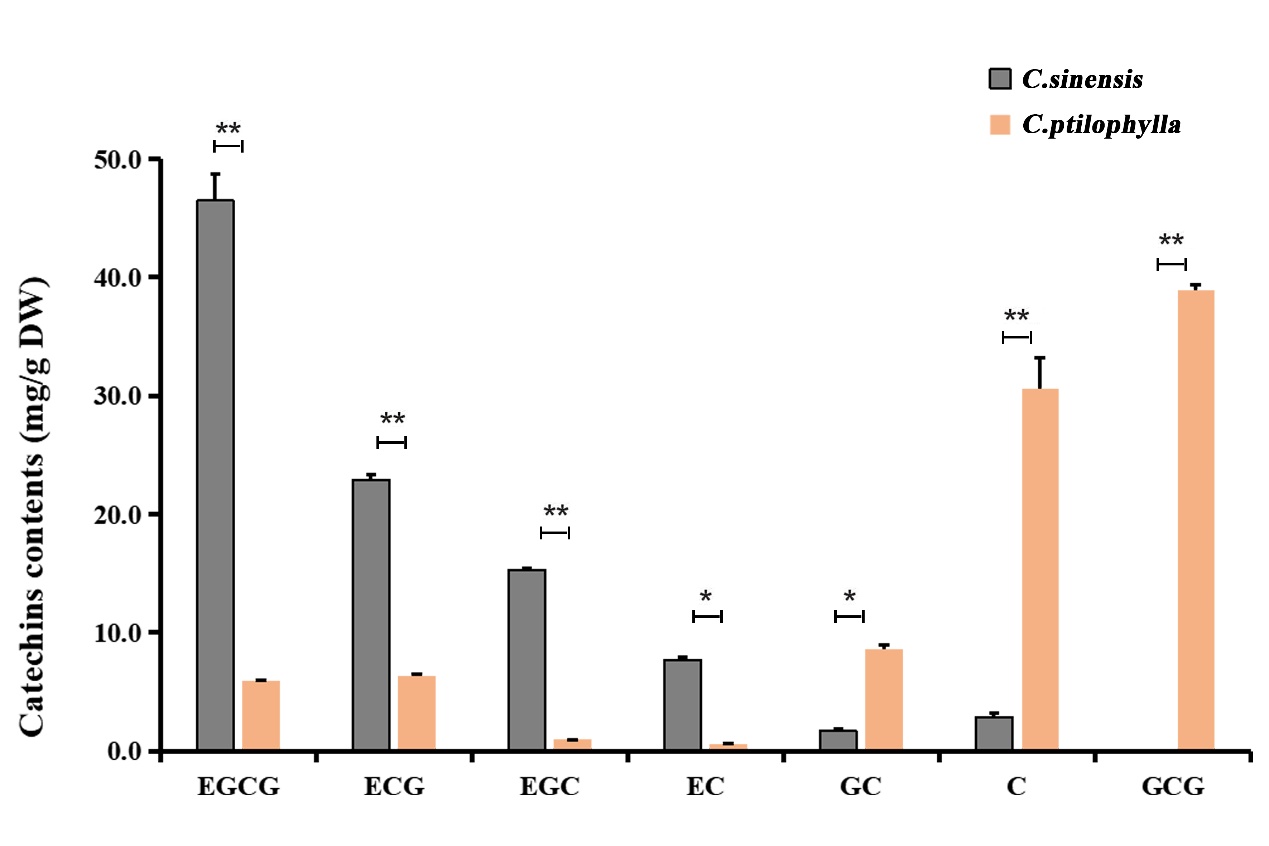


**Figure S1. Contents of catechins in leaves of** ***C.sinensis* and *C.ptilophylla***

Note: * means p<0.05, ** means p<0.01, and the statistical method is Student’s t-test


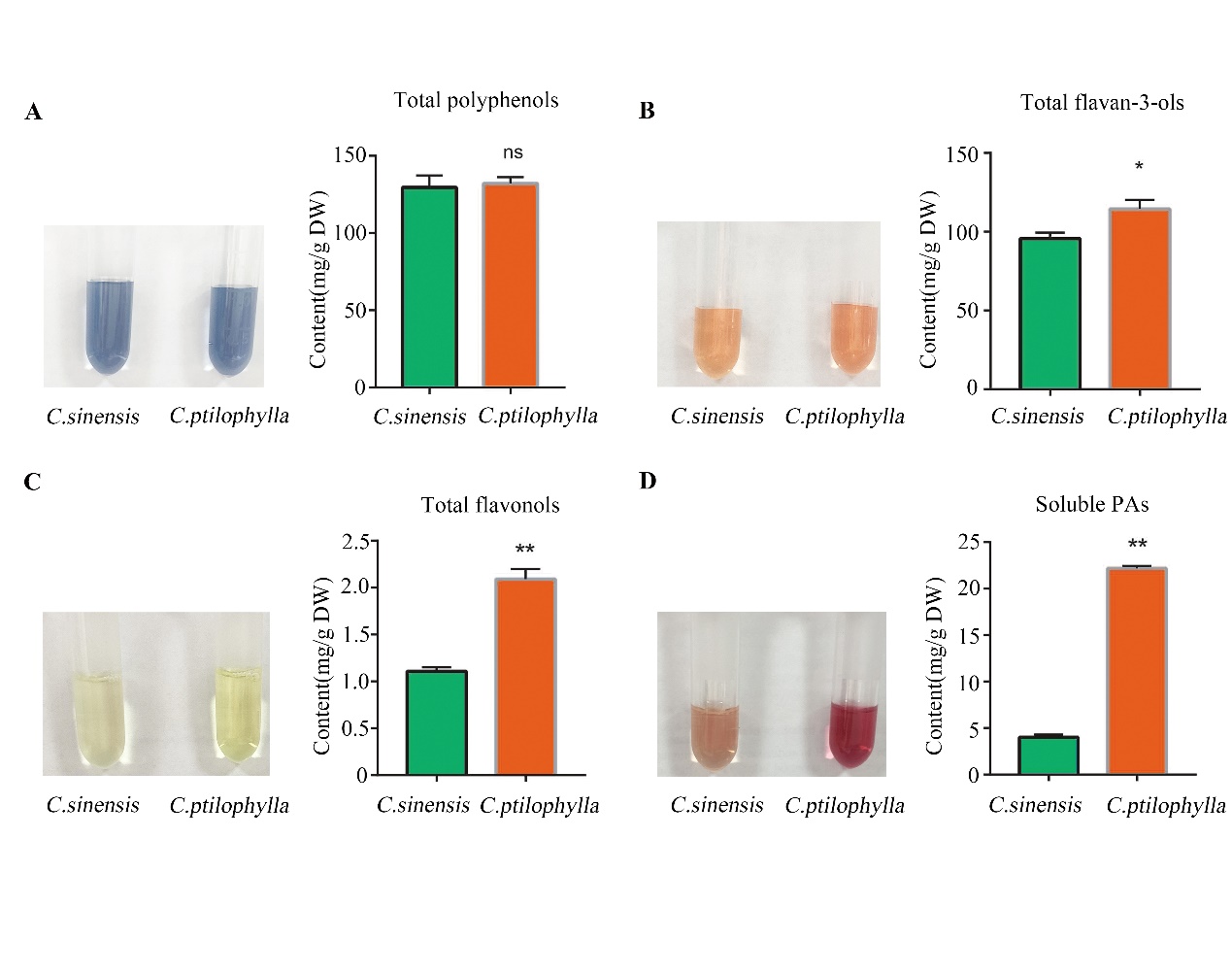


**Figure S2. Content Comparation of total polyphenol, flavan-3-ol, flavanol , flavanols and soluble proanthocyanidin between *C.ptilophylla* and** ***C.sinensis* leaves**

Note: A. The total polyphenols were measured by Folin method. B. The total flavan-3-ols was determined by the vanillin chromogenic method. C. The total flavanols was measured by Aluminum salt compound method. D. Determination of soluble proanthocyanidins by n-butanollysis. ns means no significance difference, * means p<0.05, ** means p<0.01, and the statistical method is Student’s t-test


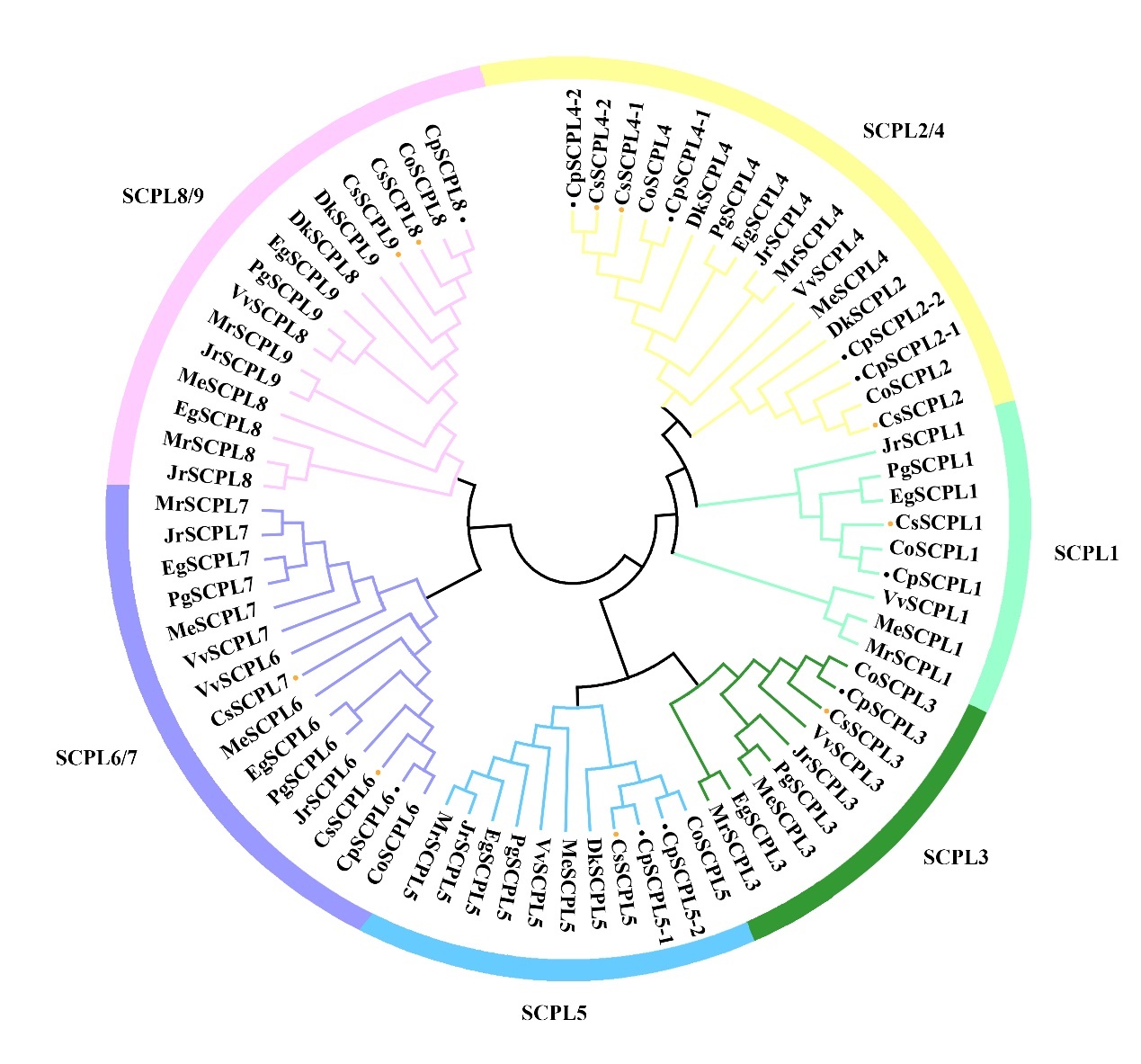


**Figure S3. Phylogenetic tree analysis of SCPL protein in *C.ptilophylla* and other species.**

The detailed protein sequence information is provided in Supplemental Data 5


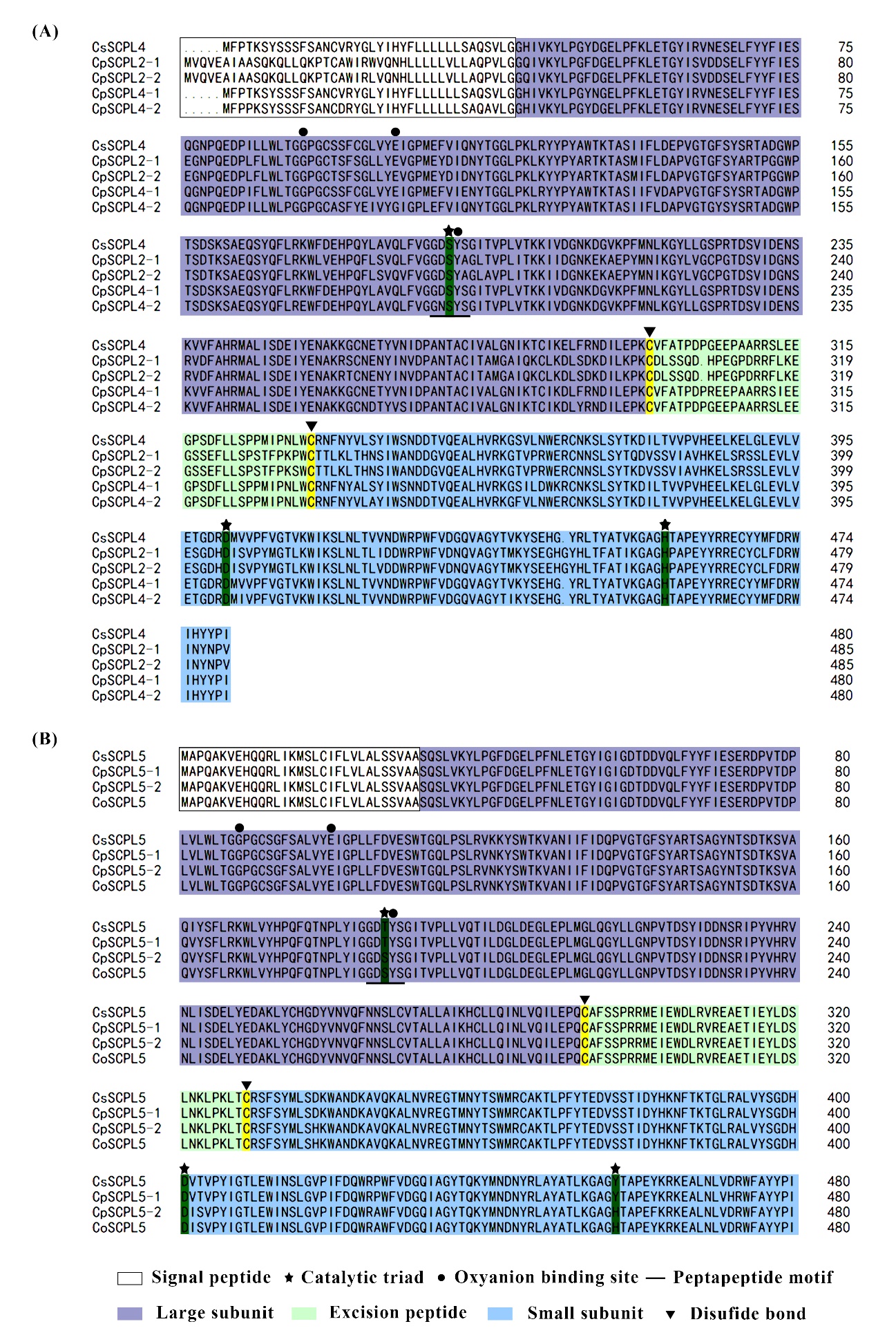


**Figure S4. Amino acid sequence alignment of SCPL4 (A) and SCPL5 (B) from *C.sinensis*, *C.ptilophylla*, and *C.oleifera***
